# Piperacillin-tazobactam-induced hemophagocytic lymphohistiocytosis in a patient with community-acquired pneumonia: A case report and literature review on diagnostic challenges of elevated procalcitonin

**DOI:** 10.1097/MD.0000000000045675

**Published:** 2025-11-21

**Authors:** Xi Xia, Di Qing, Ting Yu, JiaFu Lin, Hui Sun

**Affiliations:** a Department of Infectious Diseases, Affiliated Hospital of North Sichuan Medical College, Nanchong, Sichuan, China; b Department of General medicine, Affiliated Hospital of North Sichuan Medical College, Nanchong, Sichuan, China.

**Keywords:** adverse drug reaction, community-acquired pneumonia, hemophagocytic lymphohistiocytosis, piperacillin-tazobactam, procalcitonin

## Abstract

**Rationale:**

piperacillin-tazobactam, a widely used broad-spectrum antibiotic, carries a risk of severe adverse reactions, including rare but life-threatening hemophagocytic lymphohistiocytosis (HLH). Elevated procalcitonin (PCT), typically indicative of bacterial infection, can mask this diagnosis, leading to delayed recognition and potentially fatal outcomes. This case underscores the diagnostic challenge of drug-induced HLH mimicking infection.

**Patient concerns:**

a 17-year-old female presented with community-acquired pneumonia (CAP) and severe iron deficiency anemia. Initial piperacillin-tazobactam therapy resolved her fever and respiratory symptoms. However, after 6 afebrile days, she developed recurrent high-grade fever (40.3°C), pancytopenia (WBC 1.81 × 10^9^/L, ANC 0.23 × 10^9^/L, Hb 80 g/L, platelets 74 × 10^9^/L), hepatitis (AST 338 U/L, ALT 221 U/L), and rising serum ferritin (609.3 ng/mL) and PCT (3.057 ng/mL).

**Diagnoses:**

comprehensive evaluation excluded new infections (bacterial, viral including EBV/CMV), malignancies, autoimmune disorders, and other HLH triggers. Bone marrow morphology revealed hemophagocytic cells. Based on HLH-2004 criteria, she fulfilled 5 diagnostic criteria: fever, ≥2 lineage cytopenias, hyperferritinemia, hemophagocytosis in bone marrow, and progressive splenomegaly. The temporal association with drug exposure and resolution upon withdrawal confirmed piperacillin-tazobactam-induced HLH.

**Interventions:**

piperacillin-tazobactam was immediately discontinued upon suspicion of drug reaction. Despite elevated PCT prompting initiation of imipenem-cilastatin, the patient’s fever resolved spontaneously carbapenem before administration, and liver enzymes began improving the next day.

**Outcomes:**

following piperacillin-tazobactam cessation, fever resolved permanently within hours. Cytopenias, liver dysfunction, elevated ferritin, and PCT normalized progressively without specific HLH-directed immunosuppressive therapy. The patient was discharged symptom-free. Normal complete blood counts were confirmed at outpatient follow-ups over 13 months.

**Lessons:**

piperacillin-tazobactam can induce HLH, a critical diagnosis requiring immediate drug withdrawal. Elevated PCT in this context is a significant diagnostic pitfall, misleadingly suggesting bacterial infection progression. Unexplained fever and cytopenia during piperacillin-tazobactam therapy – even with elevated PCT – should prompt urgent evaluation for drug-induced HLH. Discontinuation of the causative agent is paramount for recovery and may obviate the need for unnecessary antimicrobial escalation or immunosuppressive therapy.

## 1. Introduction

Piperacillin-tazobactam, a β-lactam/β-lactamase inhibitor combination, exhibits broad-spectrum activity against Gram-positive, Gram-negative, and anaerobic bacteria, including *Escherichia coli* and *Pseudomonas aeruginosa*. Its efficacy against β-lactamase-producing pathogens supports widespread use amid rising antimicrobial resistance.^[1]^ Reported adverse reactions range from hematologic (e.g., leukopenia, thrombocytopenia), allergic (e.g., fever, anaphylaxis), and gastrointestinal effects to severe syndromes like drug reaction with eosinophilia and systemic symptoms (DRESS) and hemophagocytic lymphohistiocytosis (HLH).^[2–10]^

HLH is a hyperinflammatory syndrome caused by immune dysregulation, classified as primary (genetic) or secondary (triggered by infections, malignancies, autoimmune disorders, or drugs). Drug-induced HLH is rare but carries high mortality if unrecognized. Piperacillin-tazobactam has been implicated in isolated HLH cases.^[7,9,10]^ Diagnostic challenges arise when HLH mimics infection, as elevated biomarkers like procalcitonin (PCT) – a marker of bacterial sepsis – may delay recognition of drug toxicity. For instance, Song et al^[11]^ reported piperacillin-tazobactam-induced DRESS with elevated PCT, initially misinterpreted as infection progression.

We describe a 17-year-old female with community-acquired pneumonia (CAP) who developed HLH following piperacillin-tazobactam therapy. Despite elevated PCT, comprehensive evaluation excluded infection, emphasizing the need to differentiate drug-induced HLH from infectious etiologies. This case underscores vigilance for rare adverse reactions and the pivotal role of prompt drug withdrawal in managing HLH.

## 2. Case introduction

A 17-year-old female patient was admitted to the hospital due to “coughing for 14 hours, chills, and fever for 5 hours.” Around 14 hours before the admission, the patient experienced a paroxysmal dry cough without an obvious cause. Five hours before admission, she developed a fever, accompanied by chills, with a maximum body temperature of 39.5°C, fatigue, but no nausea, vomiting, abdominal distension, diarrhea, frequent urination, urgency, pain, panic, palpitations, dyspnea, dizziness, headache, or visual rotation. She visited the emergency department of our hospital and was admitted to the general medicine department on the night of November 17, 2023.

### 2.1. Past history

Diagnosed with anemia 3 years prior; received intermittent traditional Chinese medicine (details unknown), discontinued 5 months before admission.

### 2.2. Menstrual history

Irregular cycles since menarche (age 11); last menses November 8, 2023, with heavy flow (dark purple blood with clots, requiring nighttime sanitary napkin changes every 2–3 hours).

### 2.3. Physical examination

Temperature 38.8°C, pulse 122 bpm, respiration 22/min, BP 97/61 mm Hg. Pale conjunctiva noted; cardiopulmonary and abdominal examinations were unremarkable.

### 2.4. Initial investigations

**Figure 1.**
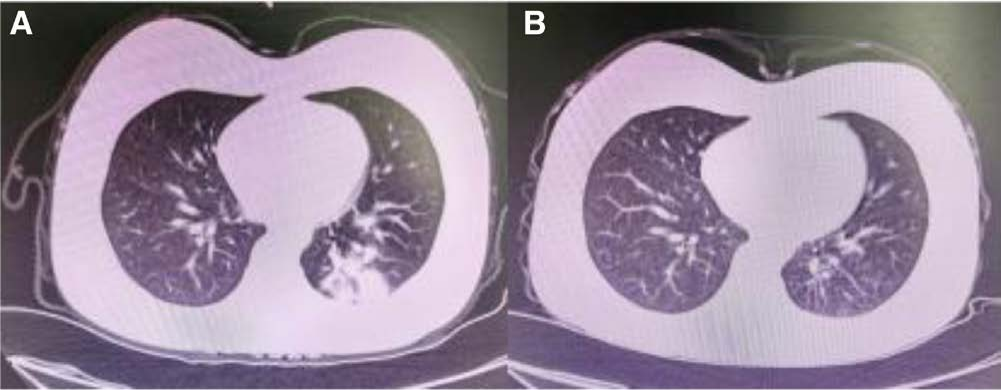
Computed tomography scan. (A) CT images before receiving anti-infection treatment with piperacillin-tazobactam. (B) CT images of patients experiencing fever again.

CBC (complete blood counts): WBC (White blood cell) 8.74 × 10^9^/L, neutrophils 79.7%, RBC (red blood cell) 3.20 × 10^12^/L, platelets 120 × 10^9^/L, hemoglobin 47 g/L.
PCT: 0.105 ng/mL.
Liver/renal function, coagulation, esophagogastroduodenoscopy (EGD) and colonoscopy: normal.
Chest CT: scattered nodular and patchy opacities in the left lower lobe (Fig. 1A).
Abdominal ultrasound: splenomegaly (13.5 cm length, 4.9 cm thickness).
Microbiology: sputum gram stain: Gram-negative bacilli; Influenza A/B viruses antigen, SARS-CoV-2 (COVID-19) nucleic acid and respiratory pathogen serology (Legionella, Adenovirus, C. pneumoniae, M. pneumoniae, RSV, parainfluenza) negative.

### 2.5. Admission diagnosis

CAP and severe anemia.

### 2.6. Treatment

Piperacillin-tazobactam (4.5 g IV q8h), polysaccharide iron complex (300 mg PO daily), Shengxuebao mixture (15 mL PO tid), ambroxol (30 mg PO tid), lansoprazole (30 mg IV daily). Anemia workup revealed iron deficiency (ferritin 4.3 ng/mL; vitamin B12 155 pg/mL; erythropoietin > 770 mIU/mL), tests for α-thalassemia, β-thalassemia, and electronic gastroenteroscopy showed no abnormalities. Hematology consultation suggested iron deficiency anemia due to heavy menstruation. Hemoglobin improved to 94 g/L posttransfusion. However, the patient was initially admitted under the care of the general medicine department. The primary antibiotic selection was made by the general practitioner, without an initial infectious disease consultation. This explains the use of piperacillin-tazobactam for CAP in this young patient.

### 2.7. Clinical course

By day 5, the patient was afebrile with improved cough. Sputum culture later grew *E coli* (amoxicillin-clavulanate MIC 16 µg/mL, intermediate), and the planned duration of antimicrobial therapy was nearing completion, Consequently, the existing regimen of piperacillin-tazobactam was continued without modification. The patient became febrile again (peak 40.3°C) after being afebrile for 6 days. Repeat chest CT showed resolving pneumonia (Fig. 1B). Investigations revealed:

Liver function: AST 338 U/L, ALT 221 U/L, ALP 152 U/L, GGT 94 U/L.
Ferritin:609.3 ng/mL.
PCT:3.057 ng/mL.
CBC: WBC 1.81 × 10^9^/L, ANC (absolute neutrophil count) 0.23 × 10^9^/L, hemoglobin 80 g/L, platelets 74 × 10^9^/L.
Abdominal CT: persistent splenomegaly (13.1 cm × 5.4 cm).

In addition to repeat thoracic and abdominal CT scans, the following investigations yielded no evidence supporting alternative infections, malignancies, or connective tissue diseases:

Anti-HAV IgM: 0.25 S/CO (reference: <1.00 S/CO).
HBsAg: <0.05 IU/mL (reference: <0.09S/CO).
Anti-HCV: 0.11 S/CO (reference: <1.00 S/CO).
Anti-HEV IgM: 0.02 S/CO (reference: <1.00 S/CO).
EBV DNA PCR:<4.0 × 10^2^ copies/mL (reference: <4.0 × 10^2^ copies/mL).
CMV DNA PCR:<4.0 × 10^2^ copies/mL (reference: <4.0 × 10^2^ copies/mL).
Blood cultures (4 bottles): no growth after 5 days (Aerobic/Anaerobic).

Transthoracic echocardiography also revealed no significant abnormalities.

#### 2.7.1. Humoral immunity assessment

Serum levels of immunoglobulin G (IgG), immunoglobulin A (IgA), immunoglobulin M (IgM), immunoglobulin E (IgE), complement C3, and complement C4 were all within the normal reference range.

#### 2.7.2. Connective tissue disease autoantibody profile

Antinuclear antibodies (ANA), anti-dsDNA (IgG) antibody, anti-Sm antibody, anti-nucleosome antibody, anti-histone antibody, anti-SSA/Ro60 antibody, anti-SSB/La antibody, anti-Ro52 antibody, anti-centromere antibody, anti-scl-70 antibody, anti-Jo-1 antibody, anti-ribosomal P protein antibody, anti-PM-Scl antibody, anti-spliceosomal complex antibody, anti-proliferating cell nuclear antigen, and anti-mitochondrial antibody (M2) were all negative/undetectable.

#### 2.7.3. Detection of anti-neutrophil cytoplasmic antibodies

The cytoplasmic pattern (c-ANCA), perinuclear pattern (p-ANCA), anti-proteinase 3 antibody (PR3-ANCA), anti-myeloperoxidase antibody (MPO-ANCA), and anti-glomerular basement membrane antibody (anti-GBM) were all negative.

#### 2.7.4. Laboratory testing for antiphospholipid syndrome (APS)

Anti-cardiolipin antibody (IgG, IgA, and IgM isotypes) and anti-β2-glycoprotein I antibody (IgG, IgA, and IgM isotypes) were within the normal range.

#### 2.7.5. Thalassemia mutation analysis

α-Thalassemia Genetic Testing (CS, QS Mutations), α-thalassemia genetic testing (WS Mutation): no mutations detected, α-thalassemia genetic testing (SEA deletion), α-thalassemia genetic testing (3.7, 4.2 deletions), β-thalassemia genetic testing (10 rare mutations including CD 27/28), and β-thalassemia genetic testing (7 Common mutations including CD 41–42) were also yielded negative results.

#### 2.7.6. Bone marrow morphological examination

**Figure 2.**
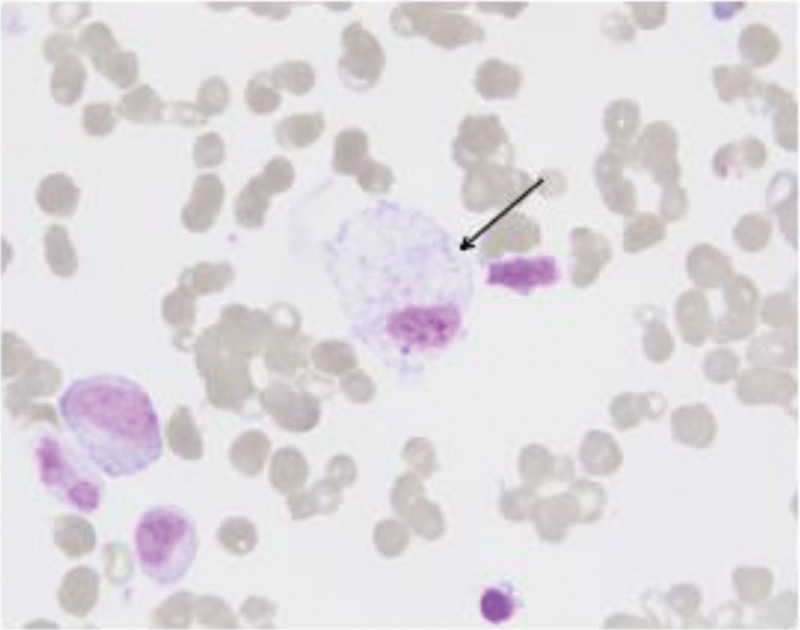
Bone marrow smear. Bone marrow smears showing activated macrophages with hemophagocytosis. Arrows indicate hemophagocytic cells phagocytosing platelets.

1. Specimen quality: adequate sampling, well-prepared smears, and good staining. small particles (+) and lipid droplets (+) are observed.
2. Bone marrow cellularity: markedly hypercellular. Granulocytic series (G) account for 60%, erythroid series (E) for 26.5%, with a G/E ratio of 2.26:1.
3. Granulocytic Series: the proportion is within normal range. some granulocytes exhibit enlarged cell bodies, vacuolization, Döhle bodies, and increased coarse cytoplasmic granules.
4. Erythroid series: increased proportion, predominantly intermediate and late-stage normoblasts. Mature red blood cells vary in size, with microcytic cells being predominant.
5. Lymphoid Series: reduced proportion, mainly consisting of mature lymphocytes. A small number of reactive lymphocytes are observed.
6. Megakaryocytes: 8 megakaryocytes are identified per entire slide. Platelets are present singly and in small clusters, easily detectable.
7. Hemophagocytosis: occasional hemophagocytic cells engulfing platelets are noted (Fig. 2).

**Figure 3.**
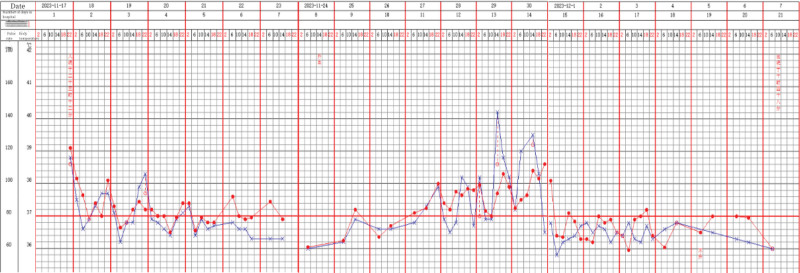
Shows the changes in the patient’s body temperature and pulse rate during hospitalization. The blue curve represents body temperature, while the red curve represents pulse rate.

Two days after the patient developed a fever again, multidisciplinary consultation involving infectious diseases, pharmacy, hematology, and rheumatology and immunology, the fever was suspected to be induced by piperacillin-tazobactam. Piperacillin-tazobactam was discontinued on the morning of the same day, but other drugs used during the same period continue to be used. Due to elevated PCT, anti-infection treatment with imipenem-cilastatin was initiated the same night. During the episode of recurrent fever, the patient was receiving piperacillin-tazobactam (for infection), ambroxol (for expectoration), mecobalamin, and nebulized acetylcysteine. Piperacillin-tazobactam was the only medication discontinued when the fever recurred, all other agents were continued. After discontinuation of piperacillin-tazobactam, the patient’s temperature has returned to normal before the addition of imipenem-cilastatint – Figure 3 shows the changes in the patient’s body temperature during hospitalization. Liver function tests performed the next day also showed improvement.

The patient continued treatment with imipenem-cilastatin for one week. Liver function, coagulation function, blood routine, ferritin, and PCT gradually returned to normal, and the patient was discharged on December 6, 2023. Due to constraints in laboratory capabilities, genetic testing could not be performed for this patient. However, follow-up CBC performed in the outpatient clinic in February 2, 2024, March 22, 2024, and January 10, 2025, all showed results within normal limits (Table 1).

**Table 1:** Follow-up complete blood count findings post-discharge.

Date	WBC	RBC	Hb	Platelets
February 2, 2024	4.83 × 10^9^/L	4.42 × 10^12^/L	125 g/L	120 × 10^9^/L
March 22, 2024	5.82 × 10^9^/L	5.04 × 10^12^/L	131 g/L	101 × 10^9^/L
January 10, 2025	7.48 × 10^9^/L	4.40 × 10^12^/L	104 g/L	124 × 10^9^/L

WBC = white blood cell, RBC = red blood cell, Hb = hemoglobin.

## 3. Discussion

According to the clinical diagnostic pathway for HLH in the Chinese Guidelines for the Diagnosis and Treatment of Hemophagocytic Syndrome (2022 Edition):

I. Identification of suspected case – triad of fever, cytopenia, and liver dysfunction: the patient presented with recurrent fever of unknown origin, accompanied by cytopenia involving all 3 hematopoietic lineages and abnormal liver function. Comprehensive investigations for common causes of liver injury and connective tissue diseases yielded unremarkable results. Furthermore, no new infectious foci or evidence of malignancy were identified. Consequently, hemophagocytic lymphohistiocytosis (HLH) was suspected.
II. Initial diagnostic step– serum ferritin: serum ferritin levels were measured in this patient and found to be ≥ 500 μg/L.
III. Confirmation of diagnosis – application of HLH-2004 diagnostic criteria^[12]^: The diagnosis of HLH requires meeting 5 out of 8 criteria. This patient fulfilled the following criteria:
  1. Fever (>38.5°C).
  2. Cytopenia involving ≥ 2 lineages (ANC: 0.23 × 10^9^/L; Hemoglobin: 80g/L; Platelets: 74 × 10^9^/L), with bone marrow morphology indicating markedly hypercellular marrow.
  3. Hyperferritinemia (serum ferritin: 609.3 ng/mL).
  4. Hemophagocytosis identified in the bone marrow (occasional phagocytosis of platelets observed).
  5. Splenomegaly, which demonstrated increased thickness compared to prior measurements following the recurrence of fever (noted present on admission but progressive).

Note: Triglyceride levels were within normal limits. Due to limited conditions, NK cell viability and sCD25 detection could not be completed.

Based on fulfillment of the above HLH-2004 diagnostic criteria, the diagnosis of hemophagocytic lymphohistiocytosis was established in this patient.

The patient initially improved with piperacillin-tazobactam, but developed a high fever again without new infection lesions or tumors, discontinuation of piperacillin-tazobactam led to normalization of her body temperature. The temporal association of fever recurred with piperacillin-tazobactam exposure, symptom resolution upon discontinuation, and exclusion of alternative triggers support drug-induced HLH. Literature reports support similar cases of hemophagocytic syndrome caused by piperacillin-tazobactam.^[7–10]^ Therefore, clinicians should consider such serious adverse reactions when patients develop fever, cytopenia during treatment with piperacillin-tazobactam.

Elevated PCT (3.057 ng/mL) during relapse initially suggested bacterial superinfection, prompting unnecessary carbapenem therapy. This mirrors Song et al’s report,^[11]^ where PCT elevation in piperacillin-tazobactam-induced DRESS delayed diagnosis. PCT, while specific for bacterial sepsis, can rise in noninfectious hyperinflammatory states like HLH. Clinicians must recognize that PCT elevation during antibiotic therapy may indicate drug toxicity rather than infection failure.

## 4. Conclusion

Piperacillin-tazobactam can induce life-threatening HLH. Elevated PCT in this context may mislead clinicians toward infectious diagnoses, delaying critical drug withdrawal. Unexplained fever and cytopenia during piperacillin-tazobactam therapy – even with elevated PCT – should prompt evaluation for HLH. Immediate cessation of the suspected drug is paramount.

Supplemental Digital Content “Supplementary File” is available for this article (https://links.lww.com/MD/Q725).

## Author contributions

**Data curation:** Xi Xia, Di Qing.

**Formal analysis:** Xi Xia, Di Qing.

**Investigation:** Xi Xia, Ting Yu, Jiafu Lin.

**Resources:** Di Qing.

**Supervision:** Hui Sun.

**Validation:** Ting Yu, Jiafu Lin, Hui Sun.

**Writing – original draft:** Xi Xia.

**Writing – review & editing:** Hui Sun.

## Supplementary Material


