# Supplementary material for: Piperacillin-tazobactam-induced hemophagocytic lymphohistiocytosis in a patient with community-acquired pneumonia: A case report and literature review on diagnostic challenges of elevated procalcitonin
Source: Medicine (Baltimore). 2025 Nov 21;104(47):e45675. doi: 10.1097/MD.0000000000045675 (PMC12643755; doi:10.1097/MD.0000000000045675)
Supplement: Supplementary file 1 [file medi-104-e45675-s001.pdf]

川北医学院附属医院检验报告单

性　别：女	科　别：赵双全/急诊内科	唯一标识：20231117G0322615
ID　号：301339636	年　龄：17岁	床　号：
标　本：全血	诊　断：*发热待诊	项　目：血常规

身份证号：51132520****1129

No	项　目	结　果		单　位	检测方法	参考范围	HR
1	白细胞	8.74		10E9/L		4.10-11.00	
2	中性粒细胞绝对值	6.95		10E9/L		1.80-8.30	
3	淋巴细胞绝对值	1.20		10E9/L		1.20-3.80	
4	单核细胞绝对值	0.54		10E9/L		0.14-0.74	
5	嗜酸性粒细胞绝对值	0.03		10E9/L		0.00-0.68	
6	嗜碱性粒细胞绝对值	0.02		10E9/L		0.00-0.07	
7	中性粒细胞百分比	79.70	↑	%		37.00-77.00	
8	淋巴细胞百分比	13.70	↓	%		17.00-54.00	
9	单核细胞百分比	6.10		%		2.00-11.00	
10	嗜酸性粒细胞百分比	0.30		%		0.00-9.00	
11	嗜碱性粒细胞百分比	0.20		%		0.00-1.00	
12	红细胞	3.20	↓	10E12/L		4.10-5.30	
13	血红蛋白	47	↓	g/L		129-172	
14	红细胞比积	0.188	↓			0.360-0.470	
15	平均红细胞体积	58.90	↓	fl		80.00-100.00	
16	平均红细胞血红蛋白含量	14.80	↓	pg		25.00-34.00	
17	平均红细胞血红蛋白浓度	251.0	↓	g/L		310-355	
18	红细胞分布宽度变异	24.90	↑	%		10.10-16.00	
19	血小板	120		10E9/L		100-407	
20	平均血小板体积	10.20		fl		8.50-11.80	
21	血小板比积	0.123				0.114-0.282	
22	血小板分布宽度	14.30		%		10.00-30.00	

HR标识为川渝检验互认项目，本检验结果仅反映送检标本的情况

采样时间：2023/11/17 21:56　　采样人员：田微

收样时间：2023/11/17 22:08　　检 验 者：李婕妤

报告时间：2023/11/17 22:28　　审 核 者：晏波　晏波

检验地址：四川省南充市顺庆区茂源南路1号(0817-2190076)。

扫描全能王
3亿人都在用的扫描App

川北医学院附属医院检验报告单

性　别：女	科　别：肖笛/全科医学	唯一标识：20231118G0041336
ID　号：671716	年　龄：17岁	床　号：07	身份证号：51132520****1129
标　本：血清	诊　断：*发热待诊贫血待诊	项　目：PCT

No	项　目	结　果		单 位	检测方法	参考范围	HR
1	降钙素原	0.105	↑	ng/ml		0.000-0.100	

HR标识为川渝检验互认项目，本检验结果仅反映送检标本的情况

采样时间：2023/11/17 23:44　　采样人员：龙春香

收样时间：2023/11/18 00:07　　检 验 者：邓佳

报告时间：2023/11/18 11:52　　审 核 者：唐国辉

检验地址：四川省南充市顺庆区茂源南路1号(0817-2190089)。

扫描全能王
3亿人都在用的扫描App

川北医学院附属医院检验报告单

性　别：女	科　别：青管/全科医学	唯一标识：20231118G0092002
ID　号：671716	年　龄：17岁	床　号：07
身份证号：51132520****1129		
标　本：血浆	诊　断：*发热待诊贫血待诊	项　目：肝功+肾功+电解质+TC+HDLC+LDLC(VLDLC)+TG

No	项　目	结　果		单 位	检测方法	参考范围	HR
1	门冬氨酸氨基移换酶	18		U/L		10-31	
2	丙氨酸氨基移换酶	10		U/L		6-29	
3	碱性磷酸酶	74		U/L		43-130	
4	谷氨酰转肽酶	15		U/L		6-26	
5	腺苷脱氨酶	23		U/L		0-25	
6	a-L-岩藻糖苷酶	19.8		U/L		0-40	
7	5`-核苷酸酶	5		U/L		1-11	
8	胆碱酯酶	5832		U/L		4000-13000	
9	亮氨酸氨基肽酶	22		U/L		20-44	
10	前白蛋白	177.4		mg/L		150.0-400.0	
11	总蛋白	77.9		g/L		68.0-88.0	
12	白蛋白	48.2		g/L		42-56	
13	球蛋白	29.7		g/L		19.0-40.0	
14	白蛋白：球蛋白比值	1.62		.		1.20-2.50	
15	总胆汁酸	8.1		umol/L		0.0-10.0	
16	总胆红素	36.9	↑	umol/L		1.7-21.0	
17	直接胆红素	15.6	↑	umol/L		0.0-7.0	
18	间接胆红素	21.3	↑	umol/L		1.7-17.0	
19	甘油三酯	0.61		mmol/L		0.50-1.80	
20	总胆固醇	2.45	↓	mmol/L		3.10-5.72	
21	高密度脂蛋白胆固醇	1.10		mmol/L		0.90-1.60	
22	低密度脂蛋白胆固醇	1.24	↓	mmol/L		1.90-3.40	
23	极低密度脂蛋白胆固醇	0.11	↓	mmol/L		0.25-0.81	
24	钾	3.99		mmol/L		3.5-4.9	
25	钠	140.6		mmol/L		135.0-145.0	
26	氯	108.5		mmol/L		98.0-110.0	
27	总钙	2.30		mmol/L		2.11-2.52	
28	总二氧化碳	23.2		mmol/L		20.2-30.0	
29	镁	0.81		mmol/L		0.75-1.02	
30	无机磷	1.00		mmol/L		0.93-1.61	
31	乳酸	1.88		mmol/L		0.50-2.20	

HR标识为川渝检验互认项目，本检验结果仅反映送检标本的情况

采样时间：2023/11/17 23:44　　采样人员：龙春香

收样时间：2023/11/18 00:08　　检 验 者：张均

报告时间：2023/11/18 00:50　　审 核 者：陈莹　陈莹

检验地址：四川省南充市顺庆区茂源南路1号(0817-2262203)。

扫描全能王
3亿人都在用的扫描App

川北医学院附属医院检验报告单

性　别：女	科　别：青苗/全科医学	唯一标识：20231118G0092002
ID　号：671716	年　龄：17岁	床　号：07
身份证号：51132520****1129		
标　本：血浆	诊　断：*发热待诊贫血待诊	项　目：肝功+肾功+电解质+TC+HDLC+LDLC(VLDLC)+TG

No	项　目	结　果		单　位	检测方法	参考范围	HR
32	尿素	3.91		mmol/L		2.50-6.50	
33	肌酐	67.1		umol/L		39.0-76.0	
34	肾小球滤过率估算值	100.4		ml/min*1.73			
35	尿酸	413.8	↑	umol/L		150.0-370.0	

HR标识为川渝检验互认项目，本检验结果仅反映送检标本的情况

采样时间：2023/11/17 23:44　　采样人员：龙春香

收样时间：2023/11/18 00:08　　检 验 者：张均

报告时间：2023/11/18 00:50　　审 核 者：陈莹　陈莹

检验地址：四川省南充市顺庆区茂源南路1号(0817-2262203)。

CS 扫描全能王
3亿人都在用的扫描App

川北医学院附属医院检验报告单

	性　别：女	科　别：青笛/全科医学	唯一标识：20231118G0325001
ID　号：671716	年　龄：17岁	床　号：07	身份证号：51132520****1129
标　本：血浆	诊　断：*发热待诊贫血待诊	项　目：D-D+术前凝血	

No	项　　目	结　果		单 位	检测方法	参考范围	HR
1	血浆凝血酶原时间	15.600	↑	s		10.000-14.000	
2	血浆凝血酶原国际标准化比率	1.32	↑			0.80-1.20	
3	血浆凝血酶原百分活动度	58.10	↓	%		70.00-130.00	
4	部份凝血活酶时间	35.80		s		24.00-39.00	
5	凝血酶时间	17.80		s		14.00-21.00	
6	血浆纤维蛋白原浓度	3.67		g/L		2.0000-4.0000	
7	纤维蛋白/纤维蛋白原降解产物含量	2.40		Ug/ml		0.00-5.00	
8	血浆抗凝血活酶III活性测定	90.90		%		80-130	
9	血浆D-二聚体	0.63		Ug/ml		0.00-1.00	

HR标识为川渝检验互认项目，本检验结果仅反映送检标本的情况

采样时间：2023/11/17 23:44　　采样人员：龙春香

收样时间：2023/11/18 00:08　　检 验 者：陈莹

报告时间：2023/11/18 00:29　　审 核 者：陈莹

检验地址：四川省南充市顺庆区茂源南路1号(0817-2190076)。

CS 扫描全能王
3亿人都在用的扫描App

川北医学院附属医院检验报告单

	性　别：女	科　别：青笛/全科医学	唯一标识：20231118G0040604
ID　号：671716	年　龄：17岁	床　号：07	身份证号：51132520****1129
标　本：血清	诊　断：*发热待诊贫血待诊	项　目：铁蛋白+EPO+VitB12+Folate	

No	项　目	结　果		单 位	检测方法	参考范围	HR
1	铁蛋白	4.30	↓	ng/mL		10-291	
2	维生素B12	155	↓	pg/mL		174-878	
3	叶酸	6.20		ng/mL		5.90-24.80	
4	促红细胞生成素	>770.00	↑	mIU/mL		2.59-18.50	

HR标识为川渝检验互认项目，本检验结果仅反映送检标本的情况

采样时间：2023/11/17 23:44　采样人员：龙春香

收样时间：2023/11/18 00:07　检 验 者：邓佳

报告时间：2023/11/18 11:24　审 核 者：唐国辉

检验地址：四川省南充市顺庆区茂源南路1号(0817-2190089)。

CS 扫描全能王
3亿人都在用的扫描App

川北医学院附属医院检验报告单

性　别：女	科　别：青笛/全科医学	唯一标识：20231118G0090401
ID　号：671716	年　龄：17岁	床　号：07
身份证号：51132520****1129		
标　本：咽拭子	诊　断：*发热待诊贫血待诊	项　目：★甲乙流抗原

No	项　目	结　果	单 位	检测方法	参考范围	HR
1	甲型流感病毒抗原	阴性		胶体金法	阴性	
2	乙型流感病毒抗原	阴性		胶体金法	阴性	

HR标识为川渝检验互认项目，本检验结果仅反映送检标本的情况

采样时间：2023/11/17 23:51　采样人员：龙春香

收样时间：2023/11/18 00:07　检 验 者：张均

报告时间：2023/11/18 00:30　审 核 者：陈莹　陈莹

检验地址：四川省南充市顺庆区茂源南路1号(0817-2262203)。

CS 扫描全能王
3亿人都在用的扫描App

川北医学院附属医院检验报告单

[illegible]	性　别：女	科　别：青留/全科医学	唯一标识：20231118G0044713
ID　号：671716	年　龄：17岁	床　号：07	身份证号：51132520****1129
标　本：血清	诊　断：*发热待诊贫血待诊	项　目：呼吸道6联检	

No	项　目	结　果	单 位	检测方法	参考范围	HR
1	嗜肺军团菌	阴性(-)			阴性(-)	
2	腺病毒抗体	阴性(-)			阴性(-)	
3	肺炎衣原体抗体IgM	阴性(-)			阴性(-)	
4	肺炎支原体抗体IgM	阴性(-)			阴性(-)	
5	副流感病毒1/2/3型抗体	阴性(-)			阴性(-)	
6	呼吸道合胞病毒抗体	阴性(-)			阴性(-)	

HR标识为川渝检验互认项目，本检验结果仅反映送检标本的情况

采样时间：2023/11/17 23:44　　采样人员：龙春香

收样时间：2023/11/18 00:08　　检 验 者：李青容

报告时间：2023/11/18 15:21　　审 核 者：汪光蓉　汪光蓉

检验地址：四川省南充市顺庆区茂源南路1号(0817-2190089)。

CS 扫描全能王
3亿人都在用的扫描App

川北医学院附属医院检验报告单

[illegible]	性　别：女	科　别：	唯一标识：*0G0312311191528
ID　号：671716	年　龄：17岁	床　号：07	身份证号：51132520****1129
标　本：痰液	诊　断：*发热待诊贫血待诊	项　目：一般细菌+抗酸+念珠	

镜检结果：查见革兰阴性杆菌1+（分布于细胞外）
镜检结果：未查见抗酸杆菌
镜检结果：鳞状上皮细胞10-25个/LPF，白细胞>25个/LPF
镜检结果：未查见念珠菌

注意：R=耐药，R* = 固定耐药，I=中介，S=敏感，SDD = 剂量依赖性敏感，WT=野生型：指菌株MIC<=ECV，说明无获得性耐药和/或突变。NWT=非野生型：指菌株MIC>ECV，则菌株有获得性和/或突变耐药。? =无参照标准

本检验结果仅反映送检标本的情况

采样时间：2023/11/18 12:26	采样人员：尹丽华
收样时间：2023/11/19 09:24	检 验 者：胡丹阳
报告时间：2023/11/19 15:19	审 核 者：郭杨柳　郭杨柳

检验地址：四川省南充市顺庆区茂源南路1号(0817-2598399)。

CS 扫描全能王
3亿人都在用的扫描App

川北医学院附属医院检验报告单

[illegible]	性　别：女	科　别：	唯一标识：*0G0312311211033
ID　号：671716	年　龄：17岁	床　号：07	身份证号：51132520****1129
标　本：痰液	诊　断：*发热待诊贫血待诊	项　目：下呼吸道一般细菌+嗜血杆菌培养及鉴定+常规药敏试验1*	

检测结果：未检出流感嗜血杆菌

药敏结果：大肠埃希菌中量生长（3+）

抗微生物药物	MIC(μg/ml)	KB(mm)	折点(S|I|R|SDD)	解释
阿米卡星(Amikacin)	<=2		16-64	敏感(S)
阿莫西林/克拉维酸 (Amoxicillin/Clavulanic acid)		16	13-18	中介(I)
氨曲南(Aztreonam)	<=1		4-16	敏感(S)
氨苄西林(Ampicillin)	4		8-32	敏感(S)
氨苄西林/舒巴坦(Ampicillin/Sulbactam)	<=2		8-32	敏感(S)
超广谱β-内酰胺酶(ESBL)	-			-
厄他培南(Ertapenem)	<=0.5		0.5-2	敏感(S)
复方新诺明 (Trimethoprim/Sulfamethoxazole)	<=1/19		2-4	敏感(S)
环丙沙星(Ciprofloxacin)	<=0.25		0.25-1	敏感(S)
庆大霉素(Gentamicin)	<=1		4-16	敏感(S)
头孢曲松(Ceftriaxone)	<=1		1-4	敏感(S)
头孢他啶(Ceftazidime)	<=1		4-16	敏感(S)
头孢替坦(Cefotetan)	<=4		16-64	敏感(S)
头孢呋辛(Cefuroxime)		23	14-18	敏感(S)
头孢吡肟(Cefepime)	<=1		2-16	敏感(S)
头孢哌酮/舒巴坦 (Cefoperazone/Sulbactam)		30	15-21	敏感(S)
头孢唑啉(Cefazolin)	>=64		2-8	耐药(R)
妥布霉素(Tobramycin)	<=1		4-16	敏感(S)
亚胺培南(Imipenem)	<=1		1-4	敏感(S)
左氧氟沙星(Levofloxacin)	<=0.25		0.5-2	敏感(S)
哌拉西林/他唑巴坦 (Piperacillin/Tazobactam)	<=4		8-32	敏感(S)

结果提示：大肠埃希菌对达托霉素、大环内酯类、夫西地酸、克林霉素、奎奴普丁/达福普汀、利福平、利奈唑胺、替考拉宁、万古霉素天然耐药。

注意：R=耐药，R* = 固定耐药，I=中介，S=敏感，SDD = 剂量依赖性敏感，WT=野生型：指菌株MIC<=ECV，说明无获得性耐药和/或突变。NWT=非野生型：指菌株MIC>ECV,则菌株有获得性和/或突变耐药。？=无参照标准

本检验结果仅反映送检标本的情况

采样时间：2023/11/18 12:26	采样人员：尹丽华
收样时间：2023/11/21 10:56	检 验 者：郭杨柳
报告时间：2023/11/24 07:53	审 核 者：刘婧　刘婧

检验地址：四川省南充市顺庆区茂源南路1号(0817-2598399)。

CS 扫描全能王
3亿人都在用的扫描App

Comprehensive Laboratory Findings During Recurrent Fever Episode

1. Complete Blood Count (CBC) with CRP

White Blood Cells (WBC):1.81 × 10^9/L (Reference: 4.10–11.00)
Neutrophils (Absolute):0.23 × 10^9/L (Reference: 1.80–8.30)
Lymphocytes (Absolute):1.25× 10^9/L (Reference: 1.20–3.80)
Monocytes (Absolute):0.20 × 10^9/L (Reference: 0.14–0.74)
Eosinophils (Absolute):0.12 × 10^9/L (Reference: 0.00–0.68)
Basophils (Absolute):0.01 × 10^9/L (Reference: 0.00–0.07)
Neutrophil Percentage:12.40.70% (Reference: 37.00–77.00)
Lymphocyte Percentage:69.50% (Reference: 17.00–54.00)
Monocyte Percentage:11.20% (Reference: 2.00–11.00)
Eosinophil Percentage: 6.40% (Reference: 0.00–9.00)
Basophil Percentage:0.50% (Reference: 0.00–1.00)
Red Blood Cells (RBC): 3.43× 10^{12}/L (Reference: 4.10–5.30)
Hemoglobin (Hb):80 g/L (Reference: 129–172)
Hematocrit (HCT):0.266 (Reference: 0.360–0.470)
Mean Corpuscular Volume (MCV):77.60 fL (Reference: 80.00–100.00)
Mean Corpuscular Hemoglobin (MCH):23.30 pg (Reference: 25.00–34.00)
Mean Corpuscular Hemoglobin Concentration (MCHC):300.0 g/L (Reference: 310–355)
Red Cell Distribution Width (RDW-CV):32.00% (Reference: 10.10–16.00)
Platelets:74× 10^9/L (Reference: 100–407)
Mean Platelet Volume (MPV):10.40 fL (Reference: 8.50–11.80)
Plateletcrit (PCT):0.077 (Reference: 0.114–0.282)
Platelet Distribution Width (PDW):14.50% (Reference: 10.00–30.00)
High-Sensitivity C-Reactive Protein (hs-CRP):36.53 mg/L (Reference: 0–5)

2. Liver Function Tests

Aspartate Aminotransferase (AST):338U/L (Reference: 10–31)
Alanine Aminotransferase (ALT):221U/L (Reference: 6–29)
Alkaline Phosphatase (ALP):152U/L (Reference: 43–130)
Gamma-Glutamyl Transferase (GGT):94 U/L (Reference: 6–26)
Adenosine Deaminase (ADA):49 U/L (Reference: 0–25)
α-L-Fucosidase (AFU):25.8 U/L (Reference: 0–40)
5'-Nucleotidase (5'-NT):43 U/L (Reference: 1–11)
Cholinesterase (CHE):6047 U/L (Reference: 4000–13000)
Leucine Aminopeptidase (LAP):58U/L (Reference: 20–44)
Prealbumin: 97.0 mg/L (Reference: 150.0–400.0)
Total Protein:75.7 g/L (Reference: 68.0–88.0)
Albumin: 43.1 g/L (Reference: 42–56)
Globulin:32.6 g/L (Reference: 19.0–40.0)
Albumin-to-Globulin Ratio (A/G):1.32 (Reference: 1.20–2.50)
Total Bile Acids (TBA):16.1 μmol/L (Reference: 0.0–10.0)
Total Bilirubin (TBIL):23.4μmol/L (Reference: 1.7–21.0)

Direct Bilirubin (DBIL):9.7μmol/L (Reference: 0.0–7.0)
Indirect Bilirubin (IBIL):13.7 μmol/L (Reference: 1.7–17.0)

3.Procalcitonin (PCT):3.057 ng/mL (Reference: 0.000–0.100)

4.Ferritin:609.30 ng/mL (Reference: 10–291)

5.Hepatitis/STI Serology:
HBsAg: <0.05 IU/mL (Reference :<0.09S/CO)
Anti-HCV Antibody: 0.11 S/CO (Reference: <1.00 S/CO)
HIV Ag/Ab:0.10 S/CO (Reference: <1.00 S/CO)
Syphilis Antibody:0.08 S/CO (Reference: <1.00 S/CO)
6.Anti-HEV IgM: 0.02 S/CO (Reference : <1.00 S/CO)

7.EBV Serology:
EBV IgM:11.20 U/mL (Reference: 0–40)
EBV Nuclear Antigen (EBNA) IgG:463.00 U/mL (Reference: 0–20)
EBV Viral Capsid Antigen (VCA) IgG:47.40 U/mL (Reference: 0–20)

8.Anti-HAV IgM: 0.25 S/CO (Reference: <1.00 S/CO)

9. EBV DNA PCR:<4.0 × 10^2 copies/mL (Reference:<4.0 × 10^2 copies/mL)
CMV DNA PCR:<4.0 × 10^2 copies/mL (Reference:<4.0 × 10^2 copies/mL)

10.Humoral immunity assessment
Immunoglobulin G (IgG) : 12.80 g/L (Reference: 7.20–15.60)
Immunoglobulin A (IgA):2000 mg/L (Reference: 800–4530)
Immunoglobulin M (IgM):1230 mg/L (Reference: 460–3040)
Immunoglobulin E (IgE): 12.10 IU/ml (Reference: 0.00–165.00)
Complement C3 (C3) : 884.0 μ mol/L(Reference: 790.0–1520.0)
Complement C4 (C4) : 256.0 μ mol/L(Reference: 160.0–380.0)

11.Connective Tissue Disease Autoantibody Profile
Antinuclear Antibodies(ANA):1:100-(Reference:1:100-)
Anti-Ro52 Antibody:<2.00RU/mL(Reference: 0.00-19.99)
Anti-Centromere Antibody:<2.00RU/mL(Reference: 0.00-19.99)
Anti-Spliceosomal Complex Antibody:<2.00RU/mL(Reference: 0.00-19.99)
Anti-Proliferating Cell Nuclear Antigen(PCNA):<2.00RU/mL(Reference: 0.00-19.99)
Anti-PM-Scl Antibody:<2.00RU/mL(Reference: 0.00-19.99)
Anti-dsDNA (IgG) Antibody:<1.00IU/mL(Reference: 0.00-9.99)
Anti-Nucleosome Antibody:<2.00RU/mL(Reference: 0.00-19.99)
Anti-Histone Antibody:<2.00RU/mL(Reference: 0.00-19.99)
Anti-SSA Antibody:<2.00RU/mL(Reference: 0.00-19.99)
Anti-SSB/La Antibody:<2.00RU/mL(Reference: 0.00-19.99)

Anti-Scl-70 Antibody:<2.00RU/mL(Reference: 0.00-19.99)
Anti-Jo-1 Antibody:<2.00RU/mL(Reference: 0.00-19.99)
Anti-Sm Antibody: <2.00RU/mL(Reference: 0.00-19.99)
Anti-Mitochondrial Antibody (M2):<2.00RU/mL(Reference: 0.00-19.99)
Anti-Ribosomal P Protein Antibody:<2.00RU/mL(Reference: 0.00-19.99)

12.Detection of Anti-Neutrophil Cytoplasmic Antibodies (ANCA)

Cytoplasmic pattern (c-ANCA):1.10-(Reference: 1.10-)
Perinuclear pattern (p-ANCA):1.10-(Reference: 1.10-)
Anti-Proteinase 3 Antibody(PR3-ANCA)<2.00RU/ml(Reference: 0.00-19.99)
Anti-MyeloperoxidaseAntibody(MPO-ANCA):<2.00RU/ml(Reference: 0.00-19.99)
Anti-Glomerular Basement Membrane Antibody (anti-GBM):<2.00RU/ml(Reference: 0.00-19.99)

13.Laboratory Testing for Antiphospholipid Syndrome (APS)

Anti-Cardiolipin Antibody,IgG:2.81 RU/mL(Reference: 0.00-19.99)
Anti-Cardiolipin Antibody,lgA:2.11RU/mL(Reference: 0.00-19.99)
Anti-Cardiolipin Antibody,lgM:9.06 RU/mL(Reference: 0.00-19.99)
Anti-β2-Glycoprotein Antibody,lgG:<2.00RU/mL(Reference: 0.00-19.99)
Anti-β2-Glycoprotein Antibody,lgA:<2.00RU/mL(Reference: 0.00-19.99)
Anti-β2-Glycoprotein Antibody,lgM:3.49RU/mL(Reference: 0.00-19.99)

14.Blood Cultures (4 bottles): No growth after 5 days (Aerobic/Anaerobic).

15.Thalassemia Mutation Analysis

α-Thalassemia Genetic Testing (CS, QS Mutations): No mutations detected.
α-Thalassemia Genetic Testing (WS Mutation): No mutations detected.
α-Thalassemia Genetic Testing (SEA Deletion): No deletion detected.
α-Thalassemia Genetic Testing (3.7, 4.2 Deletions): No deletions detected.
β-Thalassemia Genetic Testing (10 Rare Mutations including CD 27/28): No mutations detected.
β-Thalassemia Genetic Testing (7 Common Mutations including CD 41-42): No mutations detected.

Diagnosis/Interpretation & Recommendation:

Genotype: αα/αα; β/β (Wild-type for tested α- and β-globin gene loci).

No deletions or mutations were detected at the tested α- and β-globin gene loci. Clinical correlation is recommended.

16. bone marrow morphological examination

1.Specimen Quality: Adequate sampling, well-prepared smears, and good staining. Small particles (+) and lipid droplets (+) are observed.

2.Bone Marrow Cellularity: Markedly hypercellular. Granulocytic series (G) account for 60%, erythroid series (E) for 26.5%, with a G/E ratio of 2.26:1.

3.Granulocytic Series: The proportion is within normal range. Some granulocytes exhibit

enlarged cell bodies, vacuolization, Döhle bodies, and increased coarse cytoplasmic granules.

4.Erythroid Series: Increased proportion, predominantly intermediate and late-stage normoblasts. Mature red blood cells vary in size , with microcytic cells being predominant.

5.Lymphoid Series: Reduced proportion, mainly consisting of mature lymphocytes. A small number of reactive lymphocytes are observed.

6.Megakaryocytes: Eight megakaryocytes are identified per entire slide. Platelets are present singly and in small clusters, easily detectable.

7. Hemophagocytosis: Occasional hemophagocytic cells engulfing platelets are noted.

17.Respiratory Pathogen Panel:

Legionella pneumophila:Negative

Adenovirus:Negative

Mycoplasma pneumoniae IgM/IgG:Negative

Parainfluenza Virus 1/2/3:Negative

Respiratory Syncytial Virus (RSV):Negative

18.Detection of Influenza：

Detection of Influenza A Virus RNA： Negative

Detection of Influenza B Virus RNA： Negative

Consent for publication

Patient data were anonymized and no information in the masnuscript could trace back to the individual in question. And the patients' sign a research informed consent form before conducting the study.

Mar 19, 2025

Dear Editor,

We would like to submit the enclosed manuscript entitled "Piperacillin-Tazobactam-Induced Hemophagocytic Lymphohistiocytosis in a Community-Acquired Pneumonia Patient: A Case Report and Literature Review on Diagnostic Challenges of Elevated Procalcitonin", which we wish to be considered for publication in *MEDICINE*.The paper was co-authored by Xi Xia, Di Qing, Ting Yu, Jiafu Lin, Hui Sun.

In this case report,there are two main highlights of the findings.First, with the increase of drug-resistant bacteria, the use of piperacillin tazobactam is more and more widespread, with more adverse reactions, but few reports of haemphagocytic syndrome caused by it;second, the current reported cases mention no change of the PCT during the course of the patient, and the patient increased PCT when fever again, which is easy to think the infection was aggravated, but no new infection was found, and the final diagnosis of haemophagocytic syndrome. It is hoped that through this case report, the attention to the use of piperacillin tazobactam can lead to the occurrence of haemophagocytic syndrome, and the drug factors can also lead to the increase of PCT, so as to avoid the delay in diagnosis and treatment.

This manuscript has not been published or presented elsewhere.All study participants provided informed consent,and the study design was approved by the appropriate ethics review board.We have read and understood your journal’s policies,and we believe that neither the manuscript nor the study violates any of these.There are no conflicts of interest to declare.

Thank you for receiving our manuscript and considering it for publication in *MEDICINE*. We appreciate your time and look forward to your response.

Sincerely,

Xi Xia

Department of Infectious Diseases, Affiliated Hospital of North Sichuan Medical College,

Nanchong, Sichuan Province, China

Tele:0817-13990894578

E-mail:369990060@qq.com

Availability of data and materials

The data that support the findings of this study are available on request from the first author.

川北医学院附属医院检验报告单

ID 号：301339636	性 别：女	科 别：倪勋/血液一门诊
标 本：全血	年 龄：17岁	床 号：
	诊 断：*缺铁性贫血	项 目：网织红+血常规

唯一标识：20240202G0324616
身份证号：51132520****1129

No	项 目	结 果		单 位	检测方法	参考范围	HR
1	白细胞	4.83		10E9/L		4.10-11.00	
2	中性粒细胞绝对值	2.97		10E9/L		1.80-8.30	
3	淋巴细胞绝对值	1.43		10E9/L		1.20-3.80	
4	单核细胞绝对值	0.34		10E9/L		0.14-0.74	
5	嗜酸性粒细胞绝对值	0.07		10E9/L		0.00-0.68	
6	嗜碱性粒细胞绝对值	0.02		10E9/L		0.00-0.07	
7	中性粒细胞百分比	61.50		%		37.00-77.00	
8	淋巴细胞百分比	29.60		%		17.00-54.00	
9	单核细胞百分比	7.00		%		2.00-11.00	
10	嗜酸性粒细胞百分比	1.40		%		0.00-9.00	
11	嗜碱性粒细胞百分比	0.50		%		0.00-1.00	
12	红细胞	4.42		10E12/L		4.10-5.30	
13	血红蛋白	125	↓	g/L		129-172	
14	红细胞比积	0.391				0.360-0.470	
15	平均红细胞体积	88.50		fl		80.00-100.00	
16	平均红细胞血红蛋白含量	28.20		pg		25.00-34.00	
17	平均红细胞血红蛋白浓度	319.0		g/L		310-355	
18	红细胞分布宽度变异	14.00		%		10.10-16.00	
19	血小板	120		10E9/L		100-407	
20	平均血小板体积	14.20	↑	fl		8.50-11.80	
21	血小板比积	0.143				0.114-0.282	
22	血小板分布宽度	15.90		%		10.00-30.00	
23	网织红细胞计数百分比	2.260	↑	%		0.430-1.360	
24	网织红细胞计数绝对值	0.0998		10E12/L		0.0200-0.2000	
25	网织红细胞未成熟比率	10.700	↑	%		1.600-10.500	
26	网织红细胞低荧光强度比率	89.30	↓	%		89.90-98.40	
27	网织红细胞中荧光强度比率	9.60	↑	%		1.60-2.50	
28	网织红细胞高荧光强度比率	1.10		%		0.00-1.7	

HR标识为川渝检验互认项目，本检验结果仅反映送检标本的情况

采样时间：2024/02/02 11:48　采样人员：张静.
收样时间：2024/02/02 11:52　检 验 者：杨益嘉
报告时间：2024/02/02 12:14　审 核 者：张金花 张金花

检验地址：四川省南充市顺庆区茂源南路1号(0817-2190076)。

扫描全能王
3亿人都在用的扫描App

川北医学院附属医院检验报告单

性　别：女	科　别：张全波/老年科	唯一标识：20240322G0324583
ID　号：301339636	年　龄：17岁	床　号：	身份证号：51132520****1129
标　本：全血	诊　断：*特殊检查	项　目：CRP+血常规

No	项　目	结　果		单　位	检测方法	参考范围	HR
1	全血超敏C反应蛋白	1.21		mg/L		0-5	
2	白细胞	5.82		10E9/L		4.10-11.00	
3	中性粒细胞绝对值	3.95		10E9/L		1.80-8.30	
4	淋巴细胞绝对值	1.41		10E9/L		1.20-3.80	
5	单核细胞绝对值	0.37		10E9/L		0.14-0.74	
6	嗜酸性粒细胞绝对值	0.07		10E9/L		0.00-0.68	
7	嗜碱性粒细胞绝对值	0.02		10E9/L		0.00-0.07	
8	中性粒细胞百分比	67.80		%		37.00-77.00	
9	淋巴细胞百分比	24.20		%		17.00-54.00	
10	单核细胞百分比	6.40		%		2.00-11.00	
11	嗜酸性粒细胞百分比	1.20		%		0.00-9.00	
12	嗜碱性粒细胞百分比	0.40		%		0.00-1.00	
13	红细胞	5.04		10E12/L		4.10-5.30	
14	血红蛋白	131		g/L		129-172	
15	红细胞比积	0.417				0.360-0.470	
16	平均红细胞体积	82.80		fl		80.00-100.00	
17	平均红细胞血红蛋白含量	26.10		pg		25.00-34.00	
18	平均红细胞血红蛋白浓度	315.0		g/L		310-355	
19	红细胞分布宽度变异	14.90		%		10.10-16.00	
20	血小板	101		10E9/L		100-407	
21	平均血小板体积	15.60	↑	fl		8.50-11.80	
22	血小板比积	0.157				0.114-0.282	
23	血小板分布宽度	15.60		%		10.00-30.00	

HR标识为川渝检验互认项目，本检验结果仅反映送检标本的情况

采样时间：2024/03/22 09:17　　采样人员：丁丹

收样时间：2024/03/22 09:22　　检 验 者：李婕妤

报告时间：2024/03/22 10:12　　审 核 者：龙涛　龙涛

检验地址：四川省南充市顺庆区茂源南路1号(0817-2190076)。

CS 扫描全能王
3亿人都在用的扫描App

川北医学院附属医院检验报告单

[redacted]　　性　别：女　　科　别：倪勋/血液一门诊　　唯一标识：20250110G0321979

ID　号：301339636　　年　龄：18岁　　床　号：　　身份证号：51132520****1129

标　本：全血　　诊　断：缺铁性贫血　　项　目：血常规

No	项　目	结　果		单　位	检测方法	参考范围	HR
1	白细胞	7.48		10E9/L	流式法	3.50-9.50	HR3D
2	中性粒细胞绝对值	5.02		10E9/L	衍算法	1.40-7.10	
3	淋巴细胞绝对值	1.88		10E9/L	衍算法	0.70-4.75	
4	单核细胞绝对值	0.51		10E9/L	衍算法	0.10-0.60	
5	嗜酸性粒细胞绝对值	0.04		10E9/L	衍算法	0.02-0.52	
6	嗜碱性粒细胞绝对值	0.03		10E9/L	流式法	0.00-0.06	
7	中性粒细胞百分比	67.10		%	流式法	40.00-75.00	
8	淋巴细胞百分比	25.10		%	流式法	20.00-50.00	
9	单核细胞百分比	6.80		%	流式法	3.00-10.00	
10	嗜酸性粒细胞百分比	0.50		%	流式法	0.40-8.00	
11	嗜碱性粒细胞百分比	0.50		%	衍算法	0.00-1.00	
12	红细胞	4.40		10E12/L	鞘流阻抗法	3.80-5.10	HR3D
13	血红蛋白	104	↓	g/L	比色法	115-150	HR3D
14	红细胞比积	0.341	↓		衍算法	0.350-0.450	HR3D
15	平均红细胞体积	77.50	↓	fl	鞘流阻抗法	82.00-100.00	HR3D
16	平均红细胞血红蛋白含量	23.50	↓	pg	衍算法	27.00-34.00	HR3D
17	平均红细胞血红蛋白浓度	304.0	↓	g/L	衍算法	316-354	HR3D
18	红细胞分布宽度变异	19.40	↑	%	衍算法	10.10-16.00	
19	血小板	124		10E9/L	鞘流阻抗法	100-407	HR3D
20	平均血小板体积	12.20	↑	fl	衍算法	8.50-11.80	
21	血小板比积	0.151			衍算法	0.114-0.282	
22	血小板分布宽度	15.40		%	衍算法	10.00-30.00	

HR标识为川渝检验互认项目，本检验结果仅反映送检标本的情况

采样时间：2025/01/10 09:34　　采样人员：蒋娟

收样时间：2025/01/10 09:42　　检 验 者：蒋思思

报告时间：2025/01/10 10:13　　审 核 者：李君安

检验地址：四川省南充市顺庆区茂源南路1号(0817-2190076)。

扫描全能王
3亿人都在用的扫描App

Follow-up Hematological Laboratory Findings Post-Discharge

1. Complete Blood Count (CBC) with Reticulocyte Analysis

Date: 2024/02/02

White Blood Cells (WBC):4.83 × 10^9/L (Reference: 4.10–11.00)
Neutrophils (Absolute):2.97 × 10^9/L (Reference: 1.80–8.30)
Lymphocytes (Absolute):1.43 × 10^9/L (Reference: 1.20–3.80)
Monocytes (Absolute):0.34 × 10^9/L (Reference: 0.14–0.74)
Eosinophils (Absolute):0.07 × 10^9/L (Reference: 0.00–0.68)
Basophils (Absolute):0.02 × 10^9/L (Reference: 0.00–0.07)
Neutrophil Percentage:61.50% (Reference: 37.00–77.00)
Lymphocyte Percentage:29.60% (Reference: 17.00–54.00)
Monocyte Percentage:7.00% (Reference: 2.00–11.00)
Eosinophil Percentage: 1.40% (Reference: 0.00–9.00)
Basophil Percentage:0.50% (Reference: 0.00–1.00)
Red Blood Cells (RBC): 4.42 × 10^{12}/L (Reference: 4.10–5.30)
Hemoglobin (Hb):125 g/L (Reference: 129–172)
Hematocrit (HCT):0.391 (Reference: 0.360–0.470)
Mean Corpuscular Volume (MCV):88.50 fL (Reference: 80.00–100.00)
Mean Corpuscular Hemoglobin (MCH):28.20pg (Reference: 25.00–34.00)
Mean Corpuscular Hemoglobin Concentration (MCHC):319.0 g/L (Reference: 310–355)
Red Cell Distribution Width (RDW-CV):14.00% (Reference: 10.10–16.00)
Platelets:120× 10^9/L (Reference: 100–407)
Mean Platelet Volume (MPV):14.20 fL (Reference: 8.50–11.80)
Plateletcrit (PCT):0.143 (Reference: 0.114–0.282)
Platelet Distribution Width (PDW):15.90% (Reference: 10.00–30.00)
Reticulocyte Count percentage: 2.260% (Reference: 0.430–1.360)
Reticulocyte Count absolute Count:0.0998 × 10^{12}/L (Reference: 0.0200–0.2000)
Immature Reticulocyte Fraction (IRF):10.700% (Reference: 1.600–10.500)

2. Follow-up CBC with CRP

Date: 2024/03/22

White Blood Cells (WBC):5.82×10^9/L (Reference: 4.10–11.00)
Neutrophils (Absolute):3.95×10^9/L (Reference: 1.80–8.30)
Lymphocytes (Absolute):1.41×10^9/L (Reference: 1.20–3.80)
Monocytes (Absolute):0.37×10^9/L (Reference: 0.14–0.74)
Eosinophils (Absolute):0.07×10^9/L (Reference: 0.00–0.68)
Basophils (Absolute):0.02×10^9/L (Reference: 0.00–0.07)
Neutrophil Percentage:67.80% (Reference: 37.00–77.00)
Lymphocyte Percentage:24.20% (Reference: 17.00–54.00)
Monocyte Percentage:6.40% (Reference: 2.00–11.00)
Eosinophil Percentage: 1.20% (Reference: 0.00–9.00)
Basophil Percentage:0.40% (Reference: 0.00–1.00)
Red Blood Cells (RBC): 5.04×10^{12}/L (Reference: 4.10–5.30)

Hemoglobin (Hb):131 g/L (Reference: 129–172)
Hematocrit (HCT):0.417 (Reference: 0.360–0.470)
Mean Corpuscular Volume (MCV):82.80 fL (Reference: 80.00–100.00)
Mean Corpuscular Hemoglobin (MCH):26.10pg (Reference: 25.00–34.00)
Mean Corpuscular Hemoglobin Concentration (MCHC):315.0 g/L (Reference: 310–355)
Red Cell Distribution Width (RDW-CV):14.90% (Reference: 10.10–16.00)
Platelets:101×10^9/L (Reference: 100–407)
Mean Platelet Volume (MPV):15.60 fL (Reference: 8.50–11.80)
Plateletcrit (PCT):0.157(Reference: 0.114–0.282)
Platelet Distribution Width (PDW):15.60% (Reference: 10.00–30.00)
High-Sensitivity CRP (hs-CRP):1.21 mg/L (Reference: 0–5)

3. Latest CBC

Date: 2025/01/10

White Blood Cells (WBC):7.48×10^9/L (Reference: 4.10–11.00)
Neutrophils (Absolute):5.02×10^9/L (Reference: 1.80–8.30)
Lymphocytes (Absolute):1.88×10^9/L (Reference: 1.20–3.80)
Monocytes (Absolute):0.51×10^9/L (Reference: 0.14–0.74)
Eosinophils (Absolute):0.04×10^9/L (Reference: 0.00–0.68)
Basophils (Absolute):0.03×10^9/L (Reference: 0.00–0.07)
Neutrophil Percentage:67.10% (Reference: 37.00–77.00)
Lymphocyte Percentage:25.10% (Reference: 17.00–54.00)
Monocyte Percentage:6.80% (Reference: 2.00–11.00)
Eosinophil Percentage: 0.50% (Reference: 0.00–9.00)
Basophil Percentage:0.50% (Reference: 0.00–1.00)
Red Blood Cells (RBC): 4.40×10^{12}/L (Reference: 4.10–5.30)
Hemoglobin (Hb):104 g/L (Reference: 129–172)
Hematocrit (HCT):0.341 (Reference: 0.360–0.470)
Mean Corpuscular Volume (MCV):77.50 fL (Reference: 80.00–100.00)
Mean Corpuscular Hemoglobin (MCH):23.50pg (Reference: 25.00–34.00)
Mean Corpuscular Hemoglobin Concentration (MCHC):304.0 g/L (Reference: 310–355)
Red Cell Distribution Width (RDW-CV):19.40% (Reference: 10.10–16.00)
Platelets:124×10^9/L (Reference: 100–407)
Mean Platelet Volume (MPV):12.20 fL (Reference: 8.50–11.80)
Plateletcrit (PCT):0.151(Reference: 0.114–0.282)
Platelet Distribution Width (PDW):15.40% (Reference: 10.00–30.00)

Funding statement

None

Comprehensive Laboratory Findings Upon Admission

1. Complete Blood Count (CBC):

White Blood Cells (WBC):8.74 × 10^9/L (Reference: 4.10–11.00)
Neutrophils (Absolute):6.95 × 10^9/L (Reference: 1.80–8.30)
Lymphocytes (Absolute):1.20 × 10^9/L (Reference: 1.20–3.80)
Monocytes (Absolute):0.54 × 10^9/L (Reference: 0.14–0.74)
Eosinophils (Absolute):0.03 × 10^9/L (Reference: 0.00–0.68)
Basophils (Absolute):0.02 × 10^9/L (Reference: 0.00–0.07)
Neutrophil Percentage:79.70% (Reference: 37.00–77.00)
Lymphocyte Percentage:13.70% (Reference: 17.00–54.00)
Monocyte Percentage:6.10% (Reference: 2.00–11.00)
Eosinophil Percentage: 0.30% (Reference: 0.00–9.00)
Basophil Percentage:0.20% (Reference: 0.00–1.00)
Red Blood Cells (RBC): 3.20 × 10^{12}/L (Reference: 4.10–5.30)
Hemoglobin (Hb):47 g/L (Reference: 129–172)
Hematocrit (HCT):0.188 (Reference: 0.360–0.470)
Mean Corpuscular Volume (MCV):58.90 fL (Reference: 80.00–100.00)
Mean Corpuscular Hemoglobin (MCH):14.80 pg (Reference: 25.00–34.00)
Mean Corpuscular Hemoglobin Concentration (MCHC):251.0 g/L (Reference: 310–355)
Red Cell Distribution Width (RDW-CV):24.90% (Reference: 10.10–16.00)
Platelets:120 × 10^9/L (Reference: 100–407)
Mean Platelet Volume (MPV):10.20 fL (Reference: 8.50–11.80)
Plateletcrit (PCT):0.123 (Reference: 0.114–0.282)
Platelet Distribution Width (PDW):14.30% (Reference: 10.00–30.00)

2. Procalcitonin (PCT):0.105 ng/mL (Reference: 0.000–0.100)

3. Liver and Renal Function Tests

Aspartate Aminotransferase (AST):18 U/L (Reference: 10–31)
Alanine Aminotransferase (ALT):10 U/L (Reference: 6–29)
Alkaline Phosphatase (ALP):74 U/L (Reference: 43–130)
Gamma-Glutamyl Transferase (GGT):15 U/L (Reference: 6–26)
Adenosine Deaminase (ADA):23 U/L (Reference: 0–25)
α-L-Fucosidase (AFU):19.8 U/L (Reference: 0–40)
5'-Nucleotidase (5'-NT):5 U/L (Reference: 1–11)
Cholinesterase (CHE):5832 U/L (Reference: 4000–13000)
Leucine Aminopeptidase (LAP):22 U/L (Reference: 20–44)
Prealbumin: 177.4 mg/L (Reference: 150.0–400.0)
Total Protein:77.9 g/L (Reference: 68.0–88.0)
Albumin: 48.2 g/L (Reference: 42–56)
Globulin:29.7 g/L (Reference: 19.0–40.0)
Albumin-to-Globulin Ratio (A/G):1.62 (Reference: 1.20–2.50)
Total Bile Acids (TBA):8.1 μmol/L (Reference: 0.0–10.0)

Total Bilirubin (TBIL):36.9 μmol/L (Reference: 1.7–21.0)
Direct Bilirubin (DBIL):15.6 μmol/L (Reference: 0.0–7.0)
Indirect Bilirubin (IBIL):21.3 μmol/L (Reference: 1.7–17.0)
Urea:3.91 mmol/L (Reference: 2.50–6.50)
Creatinine:67.1 μmol/L (Reference: 39.0–76.0)
Estimated Glomerular Filtration Rate (eGFR):100.4 mL/min/1.73m^2
Uric Acid:413.8 μmol/L (Reference: 150.0–370.0)
Lipid Profile
Triglycerides (TG):0.61 mmol/L (Reference: 0.50–1.80)
Total Cholesterol (TC):2.45 mmol/L (Reference: 3.10–5.72)
High-Density Lipoprotein Cholesterol (HDL-C):1.10 mmol/L (Reference: 0.90–1.60)
Low-Density Lipoprotein Cholesterol (LDL-C): 1.24 mmol/L (Reference: 1.90–3.40)
Very Low-Density Lipoprotein Cholesterol (VLDL-C):0.11 mmol/L (Reference: 0.25–0.81)
Electrolytes and Metabolic Panel
Potassium (K^+):3.99 mmol/L (Reference: 3.5–4.9)
Sodium (Na^+):140.6 mmol/L (Reference: 135.0–145.0)
Chloride (Cl^-):108.5 mmol/L (Reference: 98.0–110.0)
Total Calcium (Ca^{2+}):2.30 mmol/L (Reference: 2.11–2.52)
Total Carbon Dioxide (TCO_2):23.2 mmol/L (Reference: 20.2–30.0)
Magnesium (Mg^{2+}):0.81 mmol/L (Reference: 0.75–1.02)
Inorganic Phosphorus (P):1.00 mmol/L (Reference: 0.93–1.61)
Lactate:1.88 mmol/L (Reference: 0.50–2.20)

4. Coagulation Studies
Prothrombin Time (PT):15.600 s (Reference: 10.000–14.000)
International Normalized Ratio (INR):1.32 (Reference: 0.80–1.20)
Prothrombin Activity (PTA): 58.10% (Reference: 70.00–130.00)
Activated Partial Thromboplastin Time (APTT):** 35.80 s (Reference: 24.00–39.00)
Thrombin Time (TT):17.80 s (Reference: 14.00–21.00)
Fibrinogen (FIB):3.67 g/L (Reference: 2.00–4.00)
Fibrin(ogen) Degradation Products (FDP):2.40 μg/mL (Reference: 0.00–5.00)
Antithrombin III (AT-III): 90.90% (Reference: 80–130)
D-Dimer: 0.63 μg/mL (Reference: 0.00–1.00)

5. Iron Metabolism and Hematopoietic Markers
Ferritin: 4.30 ng/mL (Reference: 10–291)
Vitamin B12:155 pg/mL (Reference: 174–878)
Folate: 6.20 ng/mL (Reference: 5.90–24.80)
Erythropoietin (EPO):>770.00 mIU/mL (Reference: 2.59–18.50)

6.Influenza A/B Antigen: Negative

7.Respiratory Pathogen Panel:
Legionella pneumophila:Negative

Adenovirus:Negative

Mycoplasma pneumoniae IgM/IgG:Negative

Parainfluenza Virus 1/2/3:Negative

Respiratory Syncytial Virus (RSV):Negative

8.Sputum Microscopy and Culture:

Gram Stain: Gram-negative bacilli (1+, extracellular)

Acid-Fast Bacilli (AFB):Negative

Fungal Elements (Candida): Negative

9.Bacterial Culture:

Escherichia coli (3+ growth)

Antimicrobial Susceptibility:

	MIC（ug/ml）	KB（mm）		
Amikacin	<=2		16-64	S
Amoxicillin/Clavulanic acid		16(KB)	13-18	I
Aztrconam	<=1		4-16	S
Ampicillin	4		8-32	S
Ampicillin/Sulbactam	<=2		8-32	S
Ertapenem	<=0.5		0.5-2	S
Trimethoprim/Sulfamethoxazole	<=1/19		2-4	S
Ciprofloxacin	<=0.25		0.25-1	S
Gentamicin	<=1		4-16	S
Ceftriaxone	<=1		1-4	S
Ceftazidime	<=1		4-16	S
Cefotetan	<=4		16-64	S
Cefuroxime		23	14-18	S
Cefepime	<=1		2-16	S
Cefoperazone/Sulbactam		30	15-21	S
Cefazolin	>=64		2-8	R
Tobramycin	<=1		4-16	S
Imipencm	<=1		1-4	S
Levofloxacin	<=0.25		0.5-2	S
Piperacillin/Tazobactam	<=4		8-32	S
ESBL -				

川北医学院附属医院检验报告单

ID　号：671716	性　别：女	科　别：青笛/全科医学
标　本：全血	年　龄：17岁	床　号：07
	诊　断：*发热待诊贫血待诊	项　目：CRP+血常规

唯一标识：20231201G0324529
身份证号：51132520****1129

No	项　目	结　果		单 位	检测方法	参考范围	HR
1	全血超敏C反应蛋白	36.53	↑	mg/L		0-5	
2	白细胞	1.81	↓	10E9/L		4.10-11.00	
3	中性粒细胞绝对值	0.23	↓↓↓	10E9/L		1.80-8.30	
4	淋巴细胞绝对值	1.25		10E9/L		1.20-3.80	
5	单核细胞绝对值	0.20		10E9/L		0.14-0.74	
6	嗜酸性粒细胞绝对值	0.12		10E9/L		0.00-0.68	
7	嗜碱性粒细胞绝对值	0.01		10E9/L		0.00-0.07	
8	中性粒细胞百分比	12.40	↓	%		37.00-77.00	
9	淋巴细胞百分比	69.50	↑	%		17.00-54.00	
10	单核细胞百分比	11.20	↑	%		2.00-11.00	
11	嗜酸性粒细胞百分比	6.40		%		0.00-9.00	
12	嗜碱性粒细胞百分比	0.50		%		0.00-1.00	
13	红细胞	3.43	↓	10E12/L		4.10-5.30	
14	血红蛋白	80	↓	g/L		129-172	
15	红细胞比积	0.266	↓			0.360-0.470	
16	平均红细胞体积	77.60	↓	fl		80.00-100.00	
17	平均红细胞血红蛋白含量	23.30	↓	pg		25.00-34.00	
18	平均红细胞血红蛋白浓度	300.0	↓	g/L		310-355	
19	红细胞分布宽度变异	32.00	↑	%		10.10-16.00	
20	血小板	74	↓	10E9/L		100-407	
21	平均血小板体积	10.40		fl		8.50-11.80	
22	血小板比积	0.077	↓			0.114-0.282	
23	血小板分布宽度	14.50		%		10.00-30.00	
	[异门镜检]						
24	反应性淋巴细胞	6		%			

备注：血片已镜检复核。

HR标识为川渝检验互认项目，本检验结果仅反映送检标本的情况

采样时间：2023/12/01 06:11　　采样人员：洪发丹
收样时间：2023/12/01 07:46　　检 验 者：龙涛
报告时间：2023/12/01 09:59　　审 核 者：巫俊娇　巫俊娇

检验地址：四川省南充市顺庆区茂源南路1号(0817-2190076)。

CS 扫描全能王
3亿人都在用的扫描App

川北医学院附属医院检验报告单

性 别：女	科 别：青笛/全科医学	唯一标识：20231130G0023375
ID 号：671716	年 龄：17岁	床 号：07	身份证号：51132520****1129
标 本：血浆	诊 断：*发热待诊贫血待诊	项 目：肝功

No	项 目	结 果		单 位	检测方法	参考范围	HR
1	门冬氨酸氨基移换酶	338	↑	U/L		10-31	
2	丙氨酸氨基移换酶	221	↑	U/L		6-29	
3	碱性磷酸酶	152	↑	U/L		43-130	
4	谷氨酰转肽酶	94	↑	U/L		6-26	
5	腺苷脱氨酶	49	↑	U/L		0-25	
6	a-L-岩藻糖苷酶	25.8		U/L		0-40	
7	5`-核苷酸酶	43	↑	U/L		1-11	
8	胆碱酯酶	6047		U/L		4000-13000	
9	亮氨酸氨基肽酶	58	↑	U/L		20-44	
10	前白蛋白	97.0	↓	mg/L		150.0-400.0	
11	总蛋白	75.7		g/L		68.0-88.0	
12	白蛋白	43.1		g/L		42-56	
13	球蛋白	32.6		g/L		19.0-40.0	
14	白蛋白：球蛋白比值	1.32		.		1.20-2.50	
15	总胆汁酸	16.1	↑	umol/L		0.0-10.0	
16	总胆红素	23.4	↑	umol/L		1.7-21.0	
17	直接胆红素	9.7	↑	umol/L		0.0-7.0	
18	间接胆红素	13.7		umol/L		1.7-17.0	

HR标识为川渝检验互认项目，本检验结果仅反映送检标本的情况

采样时间：2023/11/30 13:40　　采样人员：尹丽华

收样时间：2023/11/30 13:47　　检 验 者：罗书久

报告时间：2023/11/30 14:51　　审 核 者：王黎　王黎

检验地址：四川省南充市顺庆区茂源南路1号(0817-2190083)。

扫描全能王
3亿人都在用的扫描App

川北医学院附属医院检验报告单

性 别：女　科 别：肾笛/全科医学　唯一标识：20231130G0041463

ID 号：671716　年 龄：17岁　床 号：07　身份证号：51132520****1129

标 本：血清　诊 断：*发热待诊贫血待诊　项 目：PCT

No	项 目	结 果		单 位	检测方法	参考范围	HR
1	降钙素原	3.057	↑	ng/ml		0.000-0.100	

HR标识为川渝检验互认项目，本检验结果仅反映送检标本的情况

采样时间：2023/11/30 08:56　采样人员：兰丹

收样时间：2023/11/30 09:20　检 验 者：邓佳

报告时间：2023/11/30 11:48　审 核 者：晏波

检验地址：四川省南充市顺庆区茂源南路1号(0817-2190089)。

CS 扫描全能王
3亿人都在用的扫描App

川北医学院附属医院检验报告单

[redacted]

	性 别：女	科 别：青留/全科医学	唯一标识：20231201G0042021
ID 号：671716	年 龄：17岁	床 号：07	身份证号：51132520****1129
标 本：血清	诊 断：*发热待诊贫血待诊	项 目：铁蛋白	

No	项 目	结 果		单 位	检测方法	参考范围	HR
1	铁蛋白	609.30	↑	ng/mL		10-291	

HR标识为川渝检验互认项目，本检验结果仅反映送检标本的情况

采样时间：2023/12/01 06:11　采样人员：洪发丹

收样时间：2023/12/01 07:46　检 验 者：邓佳

报告时间：2023/12/01 13:06　审 核 者：邓佳 邓佳

检验地址：四川省南充市顺庆区茂源南路1号(0817-2190089)。

扫描全能王
3亿人都在用的扫描App

川北医学院附属医院检验报告单

	性　别：女	科　别：肖笛/全科医学	唯一标识：20231118G0043028
ID　号：671716	年　龄：17岁	床　号：07	身份证号：51132520****1129
标　本：血清	诊　断：*发热待诊贫血待诊	项　目：免疫输血全套	

No	项　目	结　果		单 位	检测方法	参考范围	HR
1	乙肝表面抗原定量	<0.05	-	IU/ml		0.00-0.08	
2	乙肝表面抗体	45.250	+	mIU/mL		0.000-9.999	
3	乙肝E抗原	<0.050	-	PEI U/mL		0.000-0.100	
4	乙肝E抗体	2.23	-	S/CO		1.01-99.00	
5	乙肝核心抗体定量	3.08	-	S/CO		1.01-99.00	
6	抗丙肝抗体	0.11		S/CO		0.00-0.99	
7	艾滋病病毒抗原/抗体	0.10		S/CO		0.00-0.99	
8	梅毒总抗体定量	0.08		S/CO		0.00-0.99	

HR标识为川渝检验互认项目，本检验结果仅反映送检标本的情况

采样时间：2023/11/17 23:44　　采样人员：龙春香

收样时间：2023/11/18 00:07　　检 验 者：邓佳

报告时间：2023/11/18 13:53　　审 核 者：唐国辉

检验地址：四川省南充市顺庆区茂源南路1号(0817-2190089)。

扫描全能王
3亿人都在用的扫描App

川北医学院附属医院检验报告单

[redacted]	性　别：女	科　别：青笛/全科医学	唯一标识：20231130G0044620
ID　号：671716	年　龄：17岁	床　号：07	身份证号：51132520****1129
标　本：血浆	诊　断：*发热待诊贫血待诊	项　目：HEV-Ab	

No	项　　目	结　果	单 位	检测方法	参考范围	HR
1	抗戊型肝炎抗体IgM	0.02	S/CO		0.00-0.99	

HR标识为川渝检验互认项目，本检验结果仅反映送检标本的情况

采样时间：2023/11/30 08:56	采样人员：　兰丹
收样时间：2023/11/30 09:21	检 验 者：邓仁兵
报告时间：2023/11/30 15:13	审 核 者：何莉萍　何莉萍

检验地址：四川省南充市顺庆区茂源南路1号(0817-2190089)。

CS 扫描全能王
3亿人都在用的扫描App

川北医学院附属医院检验报告单

性　别：女	科　别：青笛/全科医学	唯一标识：*231130G00435000
ID　号：671716	年　龄：17岁	床　号：07
身份证号：51132520****1129		
标　本：血浆	诊　断：*发热待诊贫血待诊	项　目：HAVAb+EB病毒

No	项　目	结　果		单 位	检测方法	参考范围	HR
1	抗甲肝抗体IgM	0.25		Index		0.00-0.99	
2	抗EB病毒抗体IgM	11.20		U/ml		0.00-40.00	
3	EB病毒早期抗原抗体	<5.00		U/ml		0.00-39.99	
4	EB病毒核抗原抗体	463.00	↑	U/ml		0.00-20.00	
5	EB病毒衣壳抗原抗体	47.40	↑	U/ml		0.00-20.00	

HR标识为川渝检验互认项目，本检验结果仅反映送检标本的情况

采样时间：2023/11/30 08:56　　采样人员：兰丹

收样时间：2023/11/30 09:21　　检 验 者：邓仁兵

报告时间：2023/11/30 13:40　　审 核 者：晏波

检验地址：四川省南充市顺庆区茂源南路1号(0817-2190089)。

CS 扫描全能王
3亿人都在用的扫描App

川北医学院附属医院检验报告单

性　别：女	科　别：肖笛/全科医学	唯一标识：20231207G0070404
ID　号：671716　年　龄：17岁	床　号：07	身份证号：51132520****1129
标　本：血浆　诊　断：*发热待诊贫血待诊	项　目：EB病毒核酸检测+巨细胞病毒核酸检测	

No	项　　目	结　果		单 位	检测方法	参考范围	HR
1	EB病毒DNA	<4.000E+2	-	Copies/ml	实时荧光定量PCR	<4.0E+02	
2	巨细胞病毒DNA	<4.000E+2	-	Copies/ml	实时荧光定量PCR	<4.0E+02	

HR标识为川渝检验互认项目，本检验结果仅反映送检标本的情况

采样时间：2023/12/01 06:11　　采样人员：洪发丹

收样时间：2023/12/01 07:46　　检 验 者：章凤

报告时间：2023/12/07 14:30　　审 核 者：苟海梅　苟海梅

检验地址：四川省南充市顺庆区茂源南路1号(0817-2190062)。

CS 扫描全能王
3亿人都在用的扫描App

川北医学院附属医院检验报告单

性　别：女	科　别：唐小焰/全科医学	唯一标识：20231130G0043687
ID　号：671716	年　龄：17岁	床　号：07	身份证号：51132520****1129
标　本：血清	诊　断：*发热待诊贫血待诊	项　目：IgE+IgG+IgA+IgM+C3+C4

No	项　目	结　果	单 位	检测方法	参考范围	HR
1	免疫球蛋白G	12.80	g/L		7.20-15.60	
2	免疫球蛋白A	2000	mg/L		800-4530	
3	免疫球蛋白M	1230	mg/L		460-3040	
4	免疫球蛋白E	12.10	IU/ml		0.00-165.00	
5	补体3	884.0	mg/L		790.0-1520.0	
6	补体4	256.0	mg/L		160.0-380.0	

HR标识为川渝检验互认项目，本检验结果仅反映送检标本的情况

采样时间：2023/11/30 08:56　　采样人员：兰丹

收样时间：2023/11/30 09:21　　检 验 者：邓仁兵

报告时间：2023/11/30 16:34　　审 核 者：张茂鑫　张茂鑫

第 1 页 共 1 页

检验地址：四川省南充市顺庆区茂源南路1号(0817-2190089)。

CS 扫描全能王
3亿人都在用的扫描App

川北医学院附属医院检验报告单

ID 号：671716	性 别：女	科 别：唐小焰/全科医学
	年 龄：17岁	床 号：07
标 本：血清	诊 断：*发热待诊贫血待诊	项 目：结缔组织相关抗体定量

唯一标识：20231130G0044020
身份证号：51132520****1129

No	项 目	结 果	单 位	检测方法	参考范围	HR
1	抗核抗体	1:100-			1:100阴性(-)	
2	抗Ro52抗体定量	<2.00	RU/ml		0.00-19.99	
3	抗着丝点抗体定量	<2.00	RU/ml		0.00-19.99	
4	抗剪接体复合蛋白抗体定量	<2.00	RU/ml		0.00-19.99	
5	抗增值性细胞抗原抗体定量	<2.00	RU/ml		0.00-19.99	
6	抗PM-scl抗体定量	<2.00	RU/ml		0.00-19.99	
7	抗双链DNA抗体定量IgG	<1.00	IU/ml		0.00-9.99	
8	抗核小体抗体定量	<2.00	RU/ml		0.00-19.99	
9	抗组蛋白抗体定量	<2.00	RU/ml		0.00-19.99	
10	抗SS-A抗体定量	<2.00	RU/ml		0.00-19.99	
11	抗SS-B/La抗体定量	<2.00	RU/ml		0.00-19.99	
12	抗Scl-70抗体定量	<2.00	RU/ml		0.00-19.99	
13	抗Jo-1抗体定量	<2.00	RU/ml		0.00-19.99	
14	抗Sm抗体定量	<2.00	RU/ml		0.00-19.99	
15	抗线粒体2型抗体定量	<2.00	RU/ml		0.00-19.99	
16	抗核糖体P蛋白抗体定量	<2.00	RU/ml		0.00-19.99	

HR标识为川渝检验互认项目，本检验结果仅反映送检标本的情况

采样时间：2023/11/30 08:56　　采样人员：　兰丹
收样时间：2023/11/30 09:21　　检 验 者：邓仁兵
报告时间：2023/11/30 16:18　　审 核 者：张茂鑫　张茂鑫

检验地址：四川省南充市顺庆区茂源南路1号(0817-2190089)。

CS 扫描全能王
3亿人都在用的扫描App

川北医学院附属医院检验报告单

ID 号：671716	性 别：女	科 别：青笛/全科医学	唯一标识：20231201G0044422
标 本：血清	年 龄：17岁	床 号：07	身份证号：51132520****1129
	诊 断：*发热待诊贫血待诊	项 目：中性粒抗体	

No	项 目	结 果	单 位	检测方法	参考范围	HR
1	抗中性粒胞浆抗体胞质型	1:10-			1:10阴性(-)	
2	抗中性粒胞浆抗体核周型	1:10-			1:10阴性(-)	
3	抗蛋白酶3	<2.00	RU/ml		0.00-19.99	
4	抗髓过氧化物酶抗体	<2.00	RU/ml		0.00-19.99	
5	抗肾小球基底膜抗体	<2.00	RU/ml		0.00-19.99	

HR标识为川渝检验互认项目，本检验结果仅反映送检标本的情况

采样时间：2023/11/30 13:40　　采样人员：尹丽华

收样时间：2023/11/30 13:47　　检 验 者：邓仁兵

报告时间：2023/12/01 15:42　　审 核 者：蔡艳娟 蔡艳娟

检验地址：四川省南充市顺庆区茂源南路1号(0817-2190089)。

扫描全能王
3亿人都在用的扫描App

川北医学院附属医院检验报告单

	性　别：女	科　别：肾箭/全科医学	唯一标识：20231201G0044317
ID　号：671716	年　龄：17岁	床　号：07	身份证号：51132520****1129
标　本：血清	诊　断：*发热待诊贫血待诊	项　目：磷脂综合症	

No	项　目	结　果	单 位	检测方法	参考范围	HR
1	抗心磷脂抗体IgG	2.81	RU/ml		0.00-19.99	
2	抗心磷脂抗体IgA	2.11	RU/ml		0.00-19.99	
3	抗心磷脂抗体IgM	9.06	RU/ml		0.00-19.99	
4	抗β2-糖蛋白I抗体IgG	<2.00	RU/mL		0.00-19.99	
5	抗β2-糖蛋白I抗体IgA	<2.00	RU/mL		0.00-19.99	
6	抗β2-糖蛋白I抗体IgM	3.49	RU/mL		0.00-19.99	

HR标识为川渝检验互认项目，本检验结果仅反映送检标本的情况

采样时间：2023/11/30 13:40　　采样人员：尹丽华

收样时间：2023/11/30 13:47　　检 验 者：邓仁兵

报告时间：2023/12/01 12:06　　审 核 者：蔡艳娟　蔡艳娟

检验地址：四川省南充市顺庆区茂源南路1号(0817-2190089)。

CS 扫描全能王
3亿人都在用的扫描App

川北医学院附属医院检验报告单

	性　别：女	科　别：	唯一标识：*0G0312311280078
ID　号：671716	年　龄：17岁	床　号：07	身份证号：51132520****1129
标　本：血液	诊　断：*发热待诊贫血待诊	项　目：血培养瓶①	

检测结果：厌氧培养5天无菌生长

本检验结果仅反映送检标本的情况

采样时间：2023/11/28 19:01	采样人员：龙春香
收样时间：2023/11/28 22:28	检 验 者：岳瑾熙
报告时间：2023/12/03 23:05	审 核 者：郭杨柳　郭杨柳

检验地址：四川省南充市顺庆区茂源南路1号(0817-2598399)。

CS 扫描全能王
3亿人都在用的扫描App

川北医学院附属医院检验报告单

性　别：女	科　别：	唯一标识：*0G0312311280080
巾　号：671716	年　龄：17岁	床　号：07	身份证号：51132520****1129
标　本：血液	诊　断：*发热待诊贫血待诊	项　目：血培养瓶②

检测结果：需氧培养5天无菌生长

本检验结果仅反映送检标本的情况

采样时间：2023/11/28 19:01　　采样人员：龙春香

收样时间：2023/11/28 22:28　　检 验 者：岳瑾熙

报告时间：2023/12/03 22:34　　审 核 者：郭杨柳 郭杨柳

第 1 页 共 1 页

检验地址：四川省南充市顺庆区茂源南路1号(0817-2598399)。

扫描全能王
3亿人都在用的扫描App

川北医学院附属医院检验报告单

性　别：女	科　别：	唯一标识：*0G0312311280085
出 号：671716	年　龄：17岁	床　号：07
标　本：血液	诊　断：*发热待诊贫血待诊	项　目：血培养瓶③

身份证号：51132520****1129

检测结果：厌氧培养5天无菌生长

本检验结果仅反映送检标本的情况

采样时间：2023/11/28 19:01　　采样人员：龙春香

收样时间：2023/11/28 22:28　　检 验 者：岳瑾熙

报告时间：2023/12/03 22:34　　审 核 者：郭杨柳

第 1 页 共 1 页

检验地址：四川省南充市顺庆区茂源南路1号(0817-2598399)。

扫描全能王
3亿人都在用的扫描App

川北医学院附属医院检验报告单

[redacted] 性　别：女 科　别： 唯一标识：*0G0312311280076

ID　号：671716 年　龄：17岁 床　号：07 身份证号：51132520****1129

标　本：血液 诊　断：*发热待诊贫血待诊 项　目：血培养瓶④

检测结果：需氧培养5天无菌生长

本检验结果仅反映送检标本的情况

采样时间：2023/11/28 19:00 采样人员：龙春香

收样时间：2023/11/28 22:28 检 验 者：岳瑾熙

报告时间：2023/12/03 23:05 审 核 者：郭杨柳

第 1 页 共 1 页

检验地址：四川省南充市顺庆区茂源南路1号(0817-2598399)。

[redacted]

CS 扫描全能王
3亿人都在用的扫描App

川北医学院附属医院检验报告单

	性　别：女	科　别：青笛/全科医学	唯一标识：*0000G0212372917
ID　号：671716	年　龄：17岁	床　号：07	身份证号：51132520****1129
标　本：全血	诊　断：*发热待诊贫血待诊	项　目：α地中海贫血+β地中海贫血检查	

项目名称	检测方法	结果
α地中海贫血基因检查(CS,QS突变型)		未检测到突变
α地中海贫血基因检查(WS突变型)		未检测到突变
α地中海贫血基因检查(SEA缺失型)		未检测到突变
α地中海贫血基因检查(3.7,4.2缺失型)		未检测到突变
β地中海贫血基因检查(27/28型等10个罕见突变型)		未检测到突变
β地中海贫血基因检查(41-42等7种突变型)		未检测到突变

诊断及建议、评估：

基因型为：αα/αα;β/β
未查见上述α和β珠蛋白基因位点的缺失和突变，请结合临床。

解释说明：

※α-地中海贫血为常染色体隐性遗传病，是α珠蛋白基因缺失、缺陷、突变使α珠蛋白链的合成受到部分或完全抑制而引起的溶血性贫血。

※α地中海贫血缺失型实验使用GAP-PCR法检测3种常见缺失(- α3.7，-α4.2，-SEA)，本试剂验证过的基因型有(-α3.7/αα)(-α4.2/αα)(--/ αα)(-α3.7/--)(-α4.2/--)，灵敏度为100%(对22例用本方法检测为基因缺失型的标本，再用测序方法进行验证，结果一致)，特异性为100%(对50例血常规及血红蛋白电泳检测均为正常的体检标本，用本方法检测均未发现α地贫基因缺失)。

※检测结果如出现基因缺失，建议咨询遗传或临床专家。

※本实验的局限性为本方法仅检测3种中国人常见α地中海贫血缺失，但不排除其它缺失及突变的发生，余值风险为3.97%

※基因位点常用名与国际命名法的对应关系如下：--SEA(Z84721.1: g.26264_45564del 19301)-α3.7(Z84721.1:g.34164_37967del)、 -α4.2（暂无国际命名法）

※α地中海贫血突变型实验使用PCR结合反向斑点杂交法，检测3个突变点（αCS、αQS、αWS）。本试剂验证过的突变位点：αCS、αQS、αWS突变点，灵敏度为100%(对25例用本方法检测为基因突变标本，再用测序方法进行验证，结果一致)；特异性为100%（对50例血常规及血红蛋白电泳检测均为正常的体检标本，用本方法检测均未发现α地贫基因突变）。

※检测结果如出现基因缺失，建议咨询遗传或临床专家。

※本实验的局限性为本方法仅检测3种中国人常见α地中海贫血突变位点，但不排除其它缺失及突变的发生。

※基因位点常用名与国际命名法的对应关系如下：HbCS 2CD142 TAA CAA (Gln)、HbQS 2CD125 CTG CCG (Leu Pro)、HbWS 2CD122 CAC CAG (His Gln)。

※β-地中海贫血为常染色体隐性遗传病，是β珠蛋白基因突变导致β珠蛋白合成抑制而引起溶血性贫血。突变能导致β+（β-珠蛋白能合成但合成量减少）和β0（β-珠蛋白完全不能合成）。

※β-地中海贫血实验使用PCR结合反向斑点杂交法，检测17个常见突变点（β0包括Int，CD41-42，CD31，CD14-15，CD17，CD71-72，IVS-I-1，CD43，CD27/28；β+包括IVS-II-654，-28，-29，-30，-32，CAP，IVS-I-5，βE）。本试剂验证过的突变位点：CD41-42，CD14-15，IVS-II-654，-28，-29，CD71-72，CD17，βE，CAP，Int，CD27/28等常见位点，灵敏度为100%(对27例用本方法检测为基因突变标本，再用测序方法进行验证，结果一致)；特异性为100%（对74例血常规及血红蛋白电泳检测均为正常的体检标本，用本方法检测均未发现β地贫基因突变）。

※检测结果如有基因突变，建议咨询遗传或临床专家。

※β-地中海贫血的局限性为本方法主要检测17个中国人常见突变位点，但不排除其它突变的发生，余值风险为2.02%。

※基因位点常用名与国际命名法对应关系如下：CD41-42(HBB:c.124_127delTTCT)、IVS-II-654(HBB:c.316_197C>T)、-28 (HBB: c.-78A>G)、CD71-72(HBB:c.216_217insA)、CD17(HBB:c.52A>T)、βE(HBB:c.79G>A)、CD31(HBB:c.94delC)、IVS-I-1 (HBB: c.92+1G>T)、CD27-28(HBB:c.84_85insC)、CD43(HBB:c.130G>T)、-32(HBB:c.-82>A)、-29(HBB:c.-79A>G)、-30(HBB:c.-80T>C)、CD14-15(HBB:c.45_46insG)、CAP(HBB:c.-11_-8delAAAC)、Int(HBB:c.2T>G)、IVS-I-5(HBB:c.92+5G>C)

HR标识为川渝检验互认项目，本检验结果仅反映送检标本的情况

采样时间：2023/11/30 08:56	采样人员：兰丹
收样时间：2023/11/30 09:20	检 验 者：王希月
报告时间：2023/12/09 09:43	审 核 者：宋琪玲

检验地址：四川省南充市顺庆区文化路68号(0817-2190067)。

CS 扫描全能王
3亿人都在用的扫描App

川北医学院附属医院检验报告单

[redacted]　　性　别：女　　科　别：青笛/全科医学　　唯一标识：*000G01312302291

ID　号：671716　　年　龄：17岁　　床　号：07　　身份证号：51132520****1129

标　本：骨髓液　　诊　断：*发热待诊贫血待诊　　项　目：骨髓涂片细胞形态+组织化学染色(POX)+组织化学染色PAS

细胞名称		血片(%)	髓片 平均值	髓片 标准差	(%)
原始细胞			0.08	±0.01	
粒细胞系统	原始粒细胞		0.64	±0.33	
粒细胞系统	早幼粒细胞		1.57	±0.60	2.0
中性粒细胞	中幼		6.49	±2.04	16.5
中性粒细胞	晚幼		7.90	±1.97	12.5
中性粒细胞	杆状核		23.72	±3.50	22.5
中性粒细胞	分叶核		9.44	±2.92	3.0
嗜酸粒细胞	中幼		0.38	±0.23	1.5
嗜酸粒细胞	晚幼		0.49	±0.32	
嗜酸粒细胞	杆状核		1.25	±0.61	1.0
嗜酸粒细胞	分叶核		0.86	±0.61	0.5
嗜碱粒细胞	中幼		0.02	±0.05	
嗜碱粒细胞	晚幼		0.06	±0.07	
嗜碱粒细胞	杆状核		0.06	±0.09	
嗜碱粒细胞	分叶核		0.03	±0.50	0.5
红细胞系统	原始红细胞		0.57	±0.30	1.0
红细胞系统	早幼红细胞		0.92	±0.41	1.5
红细胞系统	中幼红细胞		7.41	±1.91	11.5
红细胞系统	晚幼红细胞		10.75	±2.36	12.5
粒系：红系			3.00	±1.00	2.3
淋巴系统	原始淋巴细胞		0.05	±0.09	
淋巴系统	幼稚淋巴细胞		0.47	±0.84	
淋巴系统	成熟淋巴细胞		22.78	±7.04	8.0
淋巴系统	反应性淋巴细胞				1.5
单核	原始单核细胞		0.01	±0.04	
单核	幼稚单核细胞		0.14	±0.19	
单核	成熟单核细胞		3.00	±0.88	4.0
浆细胞	原始浆细胞		0.004	±0.02	
浆细胞	幼稚浆细胞		0.104	±0.16	
浆细胞	成熟浆细胞		0.71	±0.42	
其他细胞	组织细胞		0.16	±0.21	
其他细胞	吞噬细胞		0.03	±0.09	
其他细胞	组织嗜碱细胞		0.03	±0.09	
其他细胞	分类不明细胞		0.05	±0.09	
巨核细胞	原始巨核细胞		0-3		
巨核细胞	幼稚巨核细胞		0-10		
巨核细胞	颗粒巨核细胞		10-30		
巨核细胞	产板型巨核细胞		40-70		
巨核细胞	裸核型巨核细胞		0-30		
组化染色	NAP积分值		NAP阳性率		
组化染色	POX		PAS		
组化染色	细胞内铁 3		细胞外铁	弱阳性	
组化染色	NAE		NAE-NaF		
组化染色	AS-DCE				

特征及分析：

髓象

1. 取材,涂片好,染色佳。小粒（+），油滴（+）。
2. 骨髓增生明显活跃，G=60%，E=26.5%，G/E=2.26:1。
3. 粒系比例正常，部分粒细胞胞体增大，可见空泡、杜勒小体及胞浆颗粒增多增粗等现象。
4. 红系比例增高，以中晚幼红细胞为主。成熟红细胞大小不一，以小细胞为主。
5. 淋巴系比例减低，以成熟淋巴细胞为主，可见少量反应性淋巴细胞。
6. 全片见巨核细胞8个。血小板单个、小堆分布，易见。
7. 偶见噬血细胞吞噬血小板现象

意见：

此部位有核细胞增生明显活跃，为粒系比例正常伴炎性改变，红系比例增高伴缺铁骨髓象，请结合临床

本检验结果仅反映送检标本的情况

采样时间：2023/12/01 12:43　　采样人员：尹丽华

收样时间：2023/12/01 12:48　　检 验 者：李英

报告时间：2023/12/01 17:06　　审 核 者：李英　李英

检验地址：四川省南充市顺庆区茂源南路1号(0817-2190076)。

CS 扫描全能王
3亿人都在用的扫描App

川北医学院附属医院检验报告单

	性　别：女	科　别：林霞/全科医学	唯一标识：20231129G0044727
ID　号：671716	年　龄：17岁	床　号：07	身份证号：51132520****1129
标　本：血清	诊　断：*发热待诊贫血待诊	项　目：呼吸道6联检	

No	项　目	结　果	单 位	检测方法	参考范围	HR
1	嗜肺军团菌	阴性(-)			阴性(-)	
2	腺病毒抗体	阴性(-)			阴性(-)	
3	肺炎衣原体抗体IgM	阴性(-)			阴性(-)	
4	肺炎支原体抗体IgM	阴性(-)			阴性(-)	
5	副流感病毒1/2/3型抗体	阴性(-)			阴性(-)	
6	呼吸道合胞病毒抗体	阴性(-)			阴性(-)	

HR标识为川渝检验互认项目，本检验结果仅反映送检标本的情况

采样时间：2023/11/29 10:40　　采样人员：　兰丹

收样时间：2023/11/29 11:24　　检 验 者：邓仁兵

报告时间：2023/11/29 15:40　　审 核 者：蔡艳娟　蔡艳娟

检验地址：四川省南充市顺庆区茂源南路1号(0817-2190089)。

CS 扫描全能王
3亿人都在用的扫描App

川北医学院附属医院检验报告单

性　别：女	科　别：林霞/全科医学	唯一标识：20231129G0070505
ID　号：671716	年　龄：17岁	床　号：07	身份证号：51132520****1129
标　本：咽拭子	诊　断：*发热待诊贫血待诊	项　目：甲、乙型流感病毒核酸

No	项　目	结　果	单 位	检测方法	参考范围	HR
1	甲型流感病毒RNA	阴性		实时荧光PCR	阴性	
2	乙型流感病毒RNA	阴性		实时荧光PCR	阴性	

HR标识为川渝检验互认项目，本检验结果仅反映送检标本的情况

采样时间：2023/11/29 08:45　采样人员：文玉兰

收样时间：2023/11/29 09:38　检 验 者：王舒淇

报告时间：2023/11/29 13:57　审 核 者：陈莹　陈莹

检验地址：四川省南充市顺庆区茂源南路1号(0817-2190062)。

CS 扫描全能王
3亿人都在用的扫描App

Piperacillin-Tazobactam-Induced Hemophagocytic Lymphohistiocytosis in a Patient with Community-Acquired Pneumonia: A Case Report and Literature Review on Diagnostic Challenges of Elevated Procalcitonin

Xi Xia MSc[a], Di Qing MSc[b], Ting Yu MSc[a], JiaFu Lin MSc[a], Hui Sun MSc[a]

[a] Department of Infectious Diseases, Affiliated Hospital of North Sichuan Medical College,Nanchong, Sichuan, China

[b] Department of General medicine,Affiliated Hospital of North Sichuan Medical College,Nanchong, Sichuan, China

Xi Xia e-mail:369990060@qq.com

Di Qing e-mail:619019889@qq.com

Ting Yu e-mail:642139986@qq.com

JiaFu Lin e-mail:541436583@qq.com

Hui Sun e-mail:sibylsun0514@163.com

This study was approved by the Medical Ethics Committee of the Affiliated Hospital of North Sichuan Medical College.

The patients' sign a research informed consent form before conducting the study.

The authors have no funding and conflicts of interest to disclose.

Correspondence: Sun Hui, Department of Infectious Diseases, Affiliated Hospital of North Sichuan Medical College,No.1 Maoyuan South Road, Nanchong City 637000, Sichuan Province (e-mail: sibylsun0514@163.com)

Abbreviations:

HLH=Hemophagocytic lymphohistiocytosis; CAP=Community-acquired pneumonia; PCT=Procalcitonin; DRESS=Drug reaction with eosinophilia and systemic symptoms; CBC=complete blood counts;WBC=White blood cell; RBC=red blood cell; ANC=absolute neutrophil count;EGD=Esophagogastroduodenoscopy;AST=Aspartate aminotransferase; ALT=Alanine aminotransferase; GGT=Gamma-glutamyl transferase; EBV=Epstein-Barr virus; CMV=Cytomegalovirus; ANCA=Anti-neutrophil cytoplasmic antibodies;ANA=Antinuclear antibodies;

Abstract

Rationale: Piperacillin-tazobactam, a widely used broad-spectrum antibiotic, carries a risk of severe adverse reactions, including rare but life-threatening hemophagocytic lymphohistiocytosis (HLH). Elevated procalcitonin (PCT), typically indicative of bacterial infection, can mask this diagnosis, leading to delayed recognition and potentially fatal outcomes. This case underscores the diagnostic challenge of drug-induced HLH mimicking infection.

Patient Concerns: A 17-year-old female presented with community-acquired pneumonia (CAP) and severe iron-deficiency anemia. Initial piperacillin-tazobactam therapy resolved her fever and respiratory symptoms. However, after 6 afebrile days, she developed recurrent high-grade fever (40.3°C), pancytopenia (WBC 1.81 × 10^9/L, ANC 0.23 × 10^9/L, Hb 80 g/L, platelets 74 × 10^9/L), hepatitis (AST 338 U/L, ALT 221 U

/L), and rising serum ferritin (609.3 ng/mL) and PCT (3.057 ng/mL).

Diagnoses: Comprehensive evaluation excluded new infections (bacterial, viral including EBV/CMV), malignancies, autoimmune disorders, and other HLH triggers. Bone marrow morphology revealed hemophagocytic cells. Based on HLH-2004 criteria, she fulfilled 5 diagnostic criteria: fever, ≥ 2 lineage cytopenias, hyperferritinemia, hemophagocytosis in bone marrow, and progressive splenomegaly. The temporal association with drug exposure and resolution upon withdrawal confirmed piperacillin-tazobactam-induced HLH.

Interventions: Piperacillin-tazobactam was immediately discontinued upon suspicion of drug reaction. Despite elevated PCT prompting initiation of imipenem-cilastatin, the patient's fever resolved spontaneously carbapenem before administration, and liver enzymes began improving the next day.

Outcomes: Following piperacillin-tazobactam cessation, fever resolved permanently within hours. Cytopenias, liver dysfunction, elevated ferritin, and PCT normalized progressively without specific HLH-directed immunosuppressive therapy. The patient was discharged symptom-free. Normal complete blood counts were confirmed at outpatient follow-ups over 13 months.

Lessons: Piperacillin-tazobactam can induce HLH, a critical diagnosis requiring immediate drug withdrawal. Elevated PCT in this context is a significant diagnostic pitfall, misleadingly suggesting bacterial infection progression. Unexplained fever and cytopenia during piperacillin-tazobactam therapy—even with elevated PCT—should prompt urgent evaluation for drug-induced HLH. Discontinuation of the causative agent is paramount for recovery and may obviate the need for unnecessary antimicrobial escalation or immunosuppressive therapy.

Keywords: Piperacillin-tazobactam; Hemophagocytic lymphohistiocytosis; Procalcitonin; Adverse drug reaction; Community-acquired pneumonia

1.Introduction

Piperacillin-tazobactam, a β-lactam/β-lactamase inhibitor combination, exhibits broad-spectrum activity against Gram-positive, Gram-negative, and anaerobic bacteria, including *Escherichia coli* and *Pseudomonas aeruginosa*. Its efficacy against β-lactamase-producing pathogens supports widespread use amid rising antimicrobial resistance[1] . Reported adverse reactions range from hematologic (e.g., leukopenia, thrombocytopenia), allergic (e.g., fever, anaphylaxis), and gastrointestinal effects to severe syndromes like drug reaction with eosinophilia and systemic symptoms (DRESS) and hemophagocytic lymphohistiocytosis (HLH) [2-10].

HLH is a hyperinflammatory syndrome caused by immune dysregulation, classified as primary (genetic) or secondary (triggered by infections, malignancies, autoimmune disorders, or drugs) [11]. Drug-induced HLH is rare but carries high mortality if unrecognized. Piperacillin-tazobactam has been implicated in isolated HLH cases[8-10]. Diagnostic challenges arise when HLH mimics infection, as elevated biomarkers like procalcitonin (PCT)—a marker of bacterial sepsis—may delay recognition of drug toxicity. For instance, Gao et al. [12]reported piperacillin-tazobactam-induced DRESS with elevated PCT, initially misinterpreted as infection progression.

We describe a 17-year-old female with CAP who developed HLH following piperacillin-tazobactam therapy. Despite elevated PCT, comprehensive evaluation excluded infection, emphasizing the need to differentiate drug-induced HLH from infectious etiologies. This case underscores vigilance for rare adverse reactions and the pivotal role of prompt drug withdrawal in managing HLH.

2.Case Introduction

A 17-year-old female patient was admitted to the hospital due to "coughing for 14 hours, chills, and fever for 5 hours." Fourteen hours before admission, the patient experienced a paroxysmal dry cough without an obvious cause. Five hours before admission, she developed a fever, accompanied by chills, with a maximum body temperature of 39.5℃, fatigue, but no nausea, vomiting, abdominal distension, diarrhea, frequent urination, urgency, pain, panic, palpitations, dyspnea, dizziness, headache, or visual rotation. She visited the emergency department of our hospital and was admitted to the general medicine department on the night of November 17, 2023.

Past History: Diagnosed with anemia three years prior; received intermittent traditional Chinese medicine (details unknown), discontinued five months before admission.

Menstrual History: Irregular cycles since menarche (age 11); last menses November 8, 2023, with heavy flow (dark purple blood with clots, requiring nighttime sanitary napkin changes every 2–3 hours).

Physical Examination: Temperature 38.8°C, pulse 122 bpm, respiration 22/min, BP 97/61 mmHg. Pale conjunctiva noted; cardiopulmonary and abdominal examinations were unremarkable.

Initial Investigations:

CBC(complete blood counts):WBC(White blood cell) 8.74 × 10^9/L, neutrophils 79.7%, RBC(red blood cell) 3.20 × 10^{12}/L, platelets 120 × 10^9/L, hemoglobin 47 g/L.

PCT: 0.105 ng/mL.

Liver/renal function, coagulation,Esophagogastroduodenoscopy (EGD) and colonoscopy:Normal.

Chest CT:Scattered nodular and patchy opacities in the left lower lobe (Figure 1A).

Abdominal ultrasound:Splenomegaly (13.5 cm length, 4.9 cm thickness).

Microbiology:Sputum Gram stain: Gram-negative bacilli; Influenza A/B viruses antigen,SARS-CoV-2 (COVID-19) nucleic acid and respiratory pathogen serology (Legionella, Adenovirus, C. pneumoniae, M. pneumoniae, RSV, Parainfluenza) negative.

Admission Diagnosis: Community-acquired pneumonia and severe anemia.

Treatment: Piperacillin-tazobactam (4.5 g IV q8h), polysaccharide iron complex (300 mg PO daily), Shengxuebao mixture (15 mL PO tid), ambroxol (30 mg PO tid), lansoprazole (30 mg IV daily). Anemia workup revealed iron deficiency (ferritin 4.3 ng/mL; vitamin B12 155 pg/mL; erythropoietin >770 mIU/mL),tests for α-thalassemia, β-thalassemia, and electronic gastroenteroscopy showed no abnormalities. Hematology consultation suggested iron deficiency anemia due to heavy menstruation. Hemoglobin improved to 94 g/L post-transfusion.However,the patient was initially admitted u

nder the care of the general medicine department.,the primary antibiotic selection was made by the general practitioner, without an initial infectious disease consultation.This explains the use of piperacillin-tazobactam for CAP in this young patient.

Clinical Course:

By day 5, the patient was afebrile with improved cough. Sputum culture later grew *Escherichia coli* (amoxicillin-clavulanate MIC 16 μg/mL, intermediate), and the planned duration of antimicrobial therapy was nearing completion, Consequently, the existing regimen of piperacillin-tazobactam was continued without modification. The patient became febrile again(peak 40.3°C) after being afebrile for six days.Repeat chest CT showed resolving pneumonia (Figure 1B). Investigations revealed:

Liver function:AST 338 U/L, ALT 221 U/L, ALP 152 U/L, GGT 94 U/L.

Ferritin:609.3 ng/mL.

PCT:3.057 ng/mL.

CBC:WBC 1.81×10^9/L, ANC(absolute neutrophil count) 0.23×10^9/L, hemoglobin 80 g/L, platelets 74×10^9/L.

Abdominal CT:Persistent splenomegaly (13.1 cm×5.4 cm).

In addition to repeat thoracic and abdominal CT scans, the following investigations yielded no evidence supporting alternative infections, malignancies, or connective tissue diseases:

Anti-HAV IgM: 0.25 S/CO (Reference: <1.00 S/CO) .

HBsAg: <0.05 IU/mL (Reference :<0.09S/CO).

Anti-HCV : 0.11 S/CO (Reference: <1.00 S/CO) .

Anti-HEV IgM: 0.02 S/CO (Reference : <1.00 S/CO) .

EBV DNA PCR:<4.0×10^2 copies/mL (Reference:<4.0×10^2 copies/mL) .

CMV DNA PCR:<4.0×10^2 copies/mL (Reference:<4.0×10^2 copies/mL) .

Blood Cultures (4 bottles): No growth after 5 days (Aerobic/Anaerobic).

Transthoracic echocardiography also revealed no significant abnormalities.

Humoral immunity assessment：Serum levels of immunoglobulin G (IgG), immunoglobulin A (IgA), immunoglobulin M (IgM), immunoglobulin E (IgE), complement C3, and complement C4 were all within the normal reference range.

Connective Tissue Disease Autoantibody Profile：Antinuclear antibodies (ANA), anti-dsDNA (IgG) antibody, anti-Sm antibody, anti-nucleosome antibody, anti-histone antibody, anti-SSA/Ro60 antibody, anti-SSB/La antibody, anti-Ro52 antibody, anti-centromere antibody, anti-scl-70 antibody, anti-Jo-1 antibody, anti-ribosomal P protein antibody, anti-PM-Scl antibody, anti-spliceosomal complex antibody, anti-proliferating cell nuclear antigen (PCNA), and anti-mitochondrial antibody (M2) were all negative/undetectable.

Detection of Anti-Neutrophil Cytoplasmic Antibodies (ANCA)：The cytoplasmic pattern (c-ANCA), perinuclear pattern (p-ANCA), anti-proteinase 3 antibody (PR3-ANCA), anti-myeloperoxidase antibody (MPO-ANCA), and anti-glomerular basement membrane antibody (anti-GBM) were all negative.

Laboratory Testing for Antiphospholipid Syndrome (APS)：Anti-cardiolipin antibody (IgG, IgA, and IgM isotypes) and anti-β2-glycoprotein I antibody (IgG, IgA, and IgM isotypes) were within the normal range.

Thalassemia Mutation Analysis：α-Thalassemia Genetic Testing (CS, QS Mutations)、α-Thalassemia Genetic Testing (WS Mutation): No mutations detected、α-Thalassemia Genetic Testing (SEA Deletion)、α-Thalassemia Genetic Testing (3.7, 4.2 Deletions)、β-Thalassemia Genetic Testing (10 Rare Mutations including CD 27/28) andβ-Thalassemia Genetic Testing (7 Common Mutations including CD 41-42) were also yielded negative results.

Bone marrow morphological examination：

(a)Specimen Quality: Adequate sampling, well-prepared smears, and good staining. Small particles (+) and lipid droplets (+) are observed.

(b)Bone Marrow Cellularity: Markedly hypercellular. Granulocytic series (G) account for 60%, erythroid series (E) for 26.5%, with a G/E ratio of 2.26:1.

(c)Granulocytic Series: The proportion is within normal range. Some granulocytes exhibit enlarged cell bodies, vacuolization, Döhle bodies, and increased coarse cytoplasmic granules.

(d)Erythroid Series: Increased proportion, predominantly intermediate and late-stage normoblasts. Mature red blood cells vary in size , with microcytic cells being predominant.

(e)Lymphoid Series: Reduced proportion, mainly consisting of mature lymphocytes. A small number of reactive lymphocytes are observed.

(f)Megakaryocytes: Eight megakaryocytes are identified per entire slide. Platelets are present singly and in small clusters, easily detectable.

(g)Hemophagocytosis: Occasional hemophagocytic cells engulfing platelets are noted(Figures 2).

Two days after the patient developed a fever again, multidisciplinary consultation involving Infectious Diseases, Pharmacy, Hematology, and Rheumatology & Immunology, the fever was suspected to be induced by piperacillin-tazobactam.piperacillin-tazobactam was discontinued on the morning of the same day, but other drugs used during the same period continue to be used. Due to elevated PCT, anti-infection treatment with imipenem-cilastatin was initiated the same night. During the episode of recurrent fever, the patient was receiving piperacillin-tazobactam (for infection), ambroxol (for expectoration), mecobalamin, and nebulized acetylcysteine. Piperacillin-tazobactam was the only medication discontinued when the fever recurred,all other agents were continued. After discontinuation of piperacillin-tazobactam, the patient's temperature has returned to normal before the addition of imipenem-cilastatint(Figure 3 shows the changes in the patient's body temperature during hospitalization). Liver function tests performed the next day also showed improvement.

The patient continued treatment with imipenem-cilastatin for one week. Liver function, coagulation function, blood routine, ferritin, and PCT gradually returned to normal,and the patient was discharged on December 6, 2023.Due to constraints in laboratory capabilities, genetic testing could not be performed for this patient. However, follow-up CBC performed in the outpatient clinic in February 2, 2024, March 22, 2024, and January 10, 2025, all showed results within normal limits(Table 1).

3.Discussion

According to the clinical diagnostic pathway for HLH in the Chinese Guidelines for the Diagnosis and Treatment of Hemophagocytic Syndrome (2022 Edition) [11]:

I. Identification of Suspected Case – Triad of Fever, Cytopenia, and Liver Dysfu nction:The patient presented with recurrent fever of unknown origin, accompanied by cytopenia involving all three hematopoietic lineages and abnormal liver function. Co mprehensive investigations for common causes of liver injury and connective tissue di seases yielded unremarkable results. Furthermore, no new infectious foci or evidence of malignancy were identified. Consequently, hemophagocytic lymphohistiocytosis (HLH) was suspected.

II. Initial Diagnostic Step – Serum Ferritin:Serum ferritin levels were measured i n this patient and found to be ≥500 μg/L.

III. Confirmation of Diagnosis – Application of HLH-2004 Diagnostic Criteria [13]:The diagnosis of HLH requires meeting 5 out of 8 criteria. This patient fulfilled t he following criteria:

1. Fever (>38.5°C).

2. Cytopenia involving ≥2 lineages (ANC: 0.23×10^9/L; Hemoglobin: 80g/L; Pla telets: 74×10^9/L), with bone marrow morphology indicating markedly hypercellular marrow.

3. Hyperferritinemia (Serum ferritin: 609.3 ng/mL).

4. Hemophagocytosis identified in the bone marrow (occasional phagocytosis of platelets observed).

5. Splenomegaly, which demonstrated increased thickness compared to prior me asurements following the recurrence of fever (noted present on admission but progres sive).

Note:Triglyceride levels were within normal limits. Due to limited conditions, N K cell viability and sCD25 detection could not be completed.

Based on fulfillment of the above HLH-2004 diagnostic criteria, the diagnosis of hemophagocytic lymphohistiocytosis was established in this patient.

The patient initially improved with piperacillin-tazobactam, but developed a high fever again without new infection lesions or tumors, discontinuation of piperacillin-ta zobactam led to normalization of her body temperature. The temporal association of f ever recurred with piperacillin-tazobactam exposure, symptom resolution upon discon tinuation, and exclusion of alternative triggers support drug-induced HLH. Literature r eports support similar cases of hemophagocytic syndrome caused by piperacillin-tazo bactam[7-10]. Therefore, clinicians should consider such serious adverse reactions w hen patients develop fever, cytopenia during treatment with piperacillin-tazobactam.

Elevated PCT (3.057 ng/mL) during relapse initially suggested bacterial superinf ection, prompting unnecessary carbapenem therapy. This mirrors Gao et al.'s report[1 2] , where PCT elevation in piperacillin-tazobactam-induced DRESS delayed diagnosi s. PCT, while specific for bacterial sepsis, can rise in non-infectious hyperinflammato ry states like HLH. Clinicians must recognize that PCT elevation during antibiotic the rapy may indicate drug toxicity rather than infection failure.

4.Conclusion

Piperacillin-tazobactam can induce life-threatening HLH. Elevated PCT in this context may mislead clinicians toward infectious diagnoses, delaying critical drug withdrawal. Unexplained fever and cytopenia during piperacillin-tazobactam therapy—even with elevated PCT—should prompt evaluation for HLH. Immediate cessation of the suspected drug is paramount.

References

[1]Lister PD. Beta-lactamase inhibitor combinations with extended-spectrum penicillins: factors influencing antibacterial activity against enterobacteriaceae and Pseudomonas aeruginosa. Pharmacotherapy.2000;20(9 Pt 2):213S-228S.

[2]Wang PX, Qu SJ, Lin L. Analysis of 186 cases of adverse reactions of piperacillin sodium and tazobactam sodium for injection. Proc Clin Med. 2023;32(11):838-840.

[3]Hagiya H, Kokado R, Ueda A, et al. Association of Adverse Drug Events with Broad-spectrum Antibiotic Use in Hospitalized Patients: A Single-center Study. Intern Med. 2019;58(18):2621-2625.

[4]Zhang H, Yang L. Adverse reactions of piperacillin: A literature review of case reports. Open Med (Wars). 2024;19(1):20240931.

[5]González Díaz C, González Hermosa A, García-Lirio E, et al. Dress induced by piperacillin-tazobactam in a child. J Allergy Clin Immunol Pract. 2015; 3(4):615-7.

[6]Fahim S, Jain V, Victor G, et al. Piperacillin-tazobactam-induced drug hypersensitivity syndrome. Cutis. 2006; 77(6):353-7.

[7]Shen Y, Teng X, Zha L, et al. Drug-Induced Hypersensitivity Syndrome With Hemophagocytic Lymphohistiocytosis Related to Piperacillin-Tazobactam: A Case Report. J Investig Allergol Clin Immunol.2023; 33(6):493-495.

[8]Zhang SN, Chen SX, Wu M, et al. A case of hemophagocytic syndrome caused by piperacillin sodium and tazobactam sodium. Herald Med. 2023; 42(05):771-772.

[9]Miyabayashi H, Kumaki S, Sato A, et al. Onset of Hemophagocytic Lymphohistiocytosis during Piperacillin-Tazobactam Therapy in Three Children with Acute Focal Bacterial Nephritis. Tohoku J Exp Med. 2018,;245(1):55-59.

[10]Carter Febres M, Abbott J, Cipriano SD, et al. Drug reaction with eosinophilia and systemic symptoms (DRESS)-associated hemophagocytic lymphohistiocytosis (HLH), an important and underrecognized HLH mimic: A case report. Pediatr Blood Cancer.2021; 68(2):e28657.

[11]Branch of hematologist of Chinese Medical Association, Hematology Group, Society of Pediatrics, Chinese Medical Association, Chinese Federation of Experts on hemophagocytic syndrome. Chinese Guidelines for the Diagnosis and Treatment of Hemophagocytic Syndrome (2022 Edition). Nati Med J China.2022; 102(20):1492-1499.

[12]Song G, Cheng MQ, Li R, Zhang CQ, Sun P. Drug-induced hypersensitivity syndrome with high procalcitonin levels due to piperacillin/tazobactam and meropenem: A case report[J]. Front Med (Lausanne). 2022;9:951714. Published 2022 Oct 4.

[13]Henter JI, Horne A, Aricó M, et al. HLH-2004: Diagnostic and therapeutic guidelines for hemophagocytic lymphohistiocytosis. Pediatr Blood Cancer.2007;48(2):124-31.

Figure 1. Computed tomography scan.

1A: CT images before receiving anti-infection treatment with piperacillin-tazobactam.

1B: CT images of patients experiencing fever again.

Figure 2. Bone marrow smear.

Bone marrow smears showing activated macrophages with hemophagocytosis. Arrows indicate hemophagocytic cells phagocytosing platelets.

Figure 3. Shows the changes in the patient’s body temperature and pulse rate during hospitalization.The blue curve represents body temperature, while the red curve represents pulse rate.

Table 1. Follow-up Complete Blood Count Findings Post-Discharge

Declaration of interest's statement

The authors declare that they have no known competing financial interests or personal relationships that could have appeared to influence the work reported in this paper.
